# Evolutionary trends in animal ribosomal DNA loci: introduction to a new online database

**DOI:** 10.1007/s00412-017-0651-8

**Published:** 2017-11-30

**Authors:** Jana Sochorová, Sònia Garcia, Francisco Gálvez, Radka Symonová, Aleš Kovařík

**Affiliations:** 1 Institute of Biophysics, Academy of Sciences of the Czech Republic, CZ-61265 Brno, Czech Republic; 2 Institut Botànic de Barcelona (IBB-CSIC-ICUB), Passeig del Migdia s/n, 08038 Barcelona, Catalonia Spain; 3 Bioscripts—Centro de Investigación y Desarrollo de Recursos Científicos, 41012 Sevilla, Andalusia Spain; 4 Faculty of Science, University of Hradec Kralove, Hradecka 1285, CZ-50003 Hradec Kralove, Czech Republic

**Keywords:** 5S rDNA, 45S rDNA, Ribosomal RNA, Animal, Cytogenetics, Database

## Abstract

Ribosomal DNA (rDNA) loci encoding 5S and 45S (18S-5.8S-28S) rRNAs are important components of eukaryotic chromosomes. Here, we set up the animal rDNA database containing cytogenetic information about these loci in 1343 animal species (264 families) collected from 542 publications. The data are based on in situ hybridisation studies (both radioactive and fluorescent) carried out in major groups of vertebrates (fish, reptiles, amphibians, birds, and mammals) and invertebrates (mostly insects and mollusks). The database is accessible online at www.animalrdnadatabase.com. The median number of 45S and 5S sites was close to two per diploid chromosome set for both rDNAs despite large variation (1–74 for 5S and 1–54 for 45S sites). No significant correlation between the number of 5S and 45S rDNA loci was observed, suggesting that their distribution and amplification across the chromosomes follow independent evolutionary trajectories. Each group, irrespective of taxonomic classification, contained rDNA sites at any chromosome location. However, the distal and pericentromeric positions were the most prevalent (> 75% karyotypes) for 45S loci, while the position of 5S loci was more variable. We also examined potential relationships between molecular attributes of rDNA (homogenisation and expression) and cytogenetic parameters such as rDNA positions, chromosome number, and morphology.

**Electronic supplementary material:**

The online version of this article (10.1007/s00412-017-0651-8) contains supplementary material, which is available to authorized users.

## Introduction

Ribosomal DNA (rDNA) encodes the four essential genes needed for ribosome function: the 5S, 5.8S, 18S, and 28S rRNAs. They have been intensively studied at the cytogenetic and molecular levels. Probes derived from their conserved regions hybridise to chromosomes of diverged biological taxa, making rDNAs the first choice chromosome marker. This is probably the reason why a molecular cytogenetic approach became popular among (cyto)taxonomists in systematics studies. Development and widespread usage of fluorescence in situ hybridisation (FISH) techniques (Pinkel et al. 1986; Leitch et al. 1994) enabled to map rDNA loci on chromosomes of thousands of species over past decades until present. Employing rDNA-FISH may also provide information about the condensation status of rDNA chromatin representing, thus, a useful complement to molecular and cytogenetic (silver staining) expression studies.

rDNA evolves under the concept of concerted evolution (Zimmer et al. 1981; Dover 1982), a process maintaining its homogeneity and functionality. The process is believed to be mediated by homologous and non-homologous recombination and gene conversion. One puzzling feature is that despite overall sequence conservation (Averbeck and Eickbush 2005), rDNA tends to change the copy number (McTaggart et al. 2007; Wang et al. 2017) and position on chromosomes rapidly (Schubert and Wobus 1985; Dubcovsky and Dvorak 1995; Roy et al. 2005). Occasionally, studies have detected changes in the chromosomal location and size of specific rDNA arrays (loci). For example, the location of arrays differed between sibling species within the *Drosophila melanogaster* complex (Lohe and Roberts 1990). Rapid changes were also suggested in populations of the brown trout, *Salmo trutta* (Castro et al. 2001), and in the grasshoppers, *Eyprepocnemis plorans* (Cabrero et al. 2003) and *Podisma pedestris* (Veltsos et al. 2009).

**Fig. 1:**
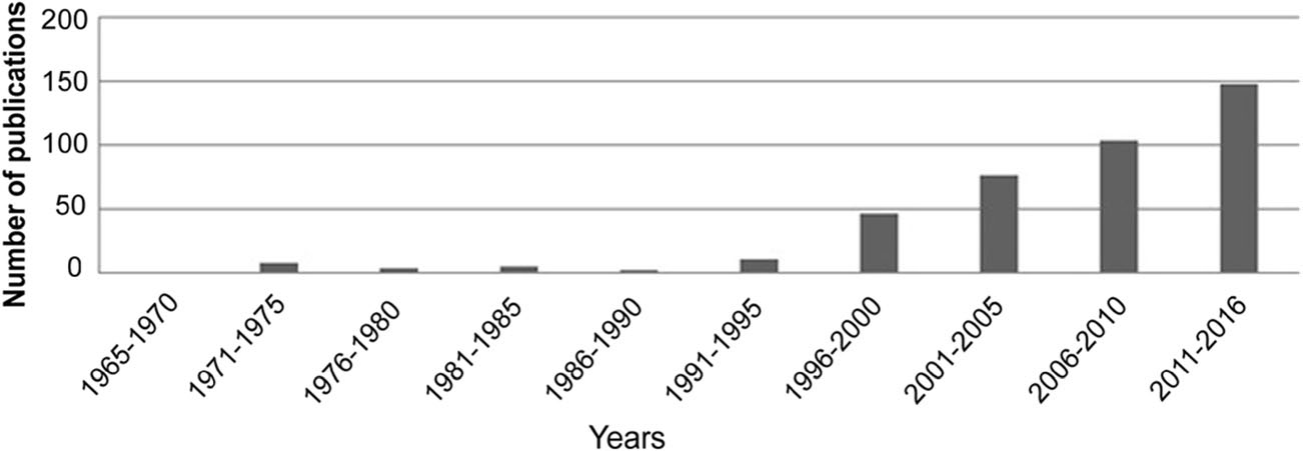
Number of publications included in the database over nine successive 5-year periods and the 6-year period 2011–2016, between 1965 and 2016

The amount of literature containing cytogenetic rDNA data has been steadily increasing in the last years (Fig. 1). For illustration purposes, the searches of WOS (Web of Science) database and Google Scholar using keywords such as “*rDNA* and *chromosome* and *in situ hybridisation* and *animal*” have yielded more than 500 results receiving annually more than 1100 citations. The literature is probably even more extensive since our search conditions were quite stringent and some works are published in non-indexed journals, conference proceedings, doctoral theses, and various monographs. Given the interest of such data through the number of publications in this area in recent times (approximately 50% of the publications listed in the database are from the last 6 years; more than 60 papers related with the topic have been published just in 2016), there is a need of assembling, storing, and analysing such information. Therefore, with the purpose of providing a tool allowing a better and easier use of animal rDNA cytogenetic information on the number and position of loci available, we have constructed the animal rDNA database. The resource is freely accessible at www.animalrdnadatabase.com representing a parallel to the plant rDNA database (www.plantrdnadatabase.com), created by our team previously (Garcia et al. 2012), providing the same information on plants. We have also analysed the database searching for relationships between the number of 5S and 45S (nucleolus organiser regions (NOR)) loci and for their preferential position (if any) on chromosomes.

## Methods

### Data assembly

The database comprises information about the number and position of rDNA in animal species collected until the end of 2016, coming from 541 publications. Papers were compiled by searching Thomson Reuters WOS, MEDLINE/PubMed, Scopus, and Google Scholar using the queries “rDNA and chromosome,” “rRNA genes and chromosome,” “rDNA and karyotype,” “rRNA and karyotype,” and “rDNA and *in situ* hybridisation.” Most journals were categorised within the areas of Genetics and Heredity, Biochemistry and Molecular Biology, Zoology, and Multidisciplinary Journals. The majority (~ 95%) of data are coming from fluorescent *in situ* hybridisation using 45S (18S, 28S, and internal transcribed spacer) and 5S rDNA probes. A smaller (~ 5%) proportion of entries was obtained from older studies based on radioactive hybridisation methods and morphological observation of secondary constrictions after the staining by classical histochemical dyes. We also aimed to include as many model representative species as possible. Together with basic information on the number, position, and linked/unlinked arrangement of rRNA genes, the resource also supplies data on chromosome number and genome size (taken from Gregory 2017) and whether the rDNA occurs on B chromosomes. The diploid locus numbers (sites) are presented as a range and mean. Three main categories of rDNA positions in chromosomes were distinguished: (i) pericentromeric = proximal sites (counting pericentromeric and centromeric positions together); (ii) distal = terminal sites (counting telomeric and subtelomeric positions together); and (iii) interstitial sites. In few cases where the hybridisation signals occupy whole chromosome arm, the database returns a “whole arm” position.

### Web site construction

The tabular database structure comprising the information on number and position of rDNA loci and the source publications was created in SQL (structured query language) tables on a MySQL server. Each table had its own different field type and size. The initial spreadsheet table in which the data were compiled was exported to a CSV (comma-separated values) file. A unique ID was given for each entry together with the date and time of the export and the version of the data. Then it was imported to the SQL database (www.animalrdnadatabase.com). The website was programmed in HTML (HyperText Markup Language), CSS (Cascading Style Sheets), and JS (Javascript) for visualisation; the custom functions in PHP (PHP: Hypertext Preprocessor) were written to query the database, process, and display the data.

### Statistical analysis

Basic statistics such as mode, average, and median were obtained through MsExcel functions. Shapiro-Wilk test for normality, Pearson’s correlation, and Mann-Whitney *U* test were performed in MsExcel and RStudio, v.0.98.1078, a user interface for R (www.rstudio.com). If a species had rDNA locus numbers that differed between or within populations, we treated each difference as a separate record. The assessment of rDNA numbers, rDNA positions, and chromosome morphologies comes exclusively from the literature, i.e. based on each authors’ evaluation, when available. In few cases where this piece of information was not explicitly mentioned in the article, the assessment was done by evaluating of provided in situ hybridisation images.

### Availability of data and materials

All data generated or analysed during this study are included in this published article, its supplementary information files, and the internet web site (http://www.animalrdnadatabase.com/).

## Results and discussion

The created database (www.animalrdnadatabase.com) includes 1358 karyotypes (Table 1). The total number of species is 1343 from eight phyla, which roughly reflect animal kingdom diversity. Yet, despite this good deal of information, much of the cytogenetic data is still missing, e.g. for model species such as *Daphnia magna* (a small planktonic crustacean) or *Columba livia* (pigeon). Surprisingly, little cytogenetic information also exists for domesticated animals such as cats and reports in *Felidae* are limited to classical karyological studies in the leopard (Tanomtong et al. 2008). Furthermore, except for a few fish (Mantovani et al. 2005) and insect (Cabrero et al. 2003) genera, the interpopulation-level studies assessing cytogenetic variability have been rarely attempted. Hence, the database content could be a useful source for further research.

**Table 1:** Species representation of the rDNA database

Taxonomy/group		Database content			Group diversity^a^
		Karyotypes		Families	
		*N*	%^b^		
Vertebrates	Actinopterygian fish	539	39.8	95	30,000
	Mammals	169	12.4	33	5500
	Amphibians	44	3.2	10	6200
	Reptiles	72	5.3	26	8200
	Lampreys	2	0.1	1	38
	Cartilaginous fish	5	0.4	3	1100
	birds	15	1.1	7	10,000
Invertebrates	Arthropods	435	32.0	57	> 1,000,000
	Mollusks	54	4.0	17	81,000
	Annelids	10	0.7	5	9000
	Flatworms	8	0.6	6	25,000
	Thorny-head-worms	2	0.1	1	1500
	Echinoderms	1	0.1	1	6000
	Nematodes	1	0.1	1	2200
	Tunicates	1	0.1	1	7000
Total		1358	100.0	264	

^a^Estimated number of species in a group. Source: http://www.encyclopedia.com/

^b^Percentage of total karyotypes

### Number of loci per karyotype

**Fig. 2:**
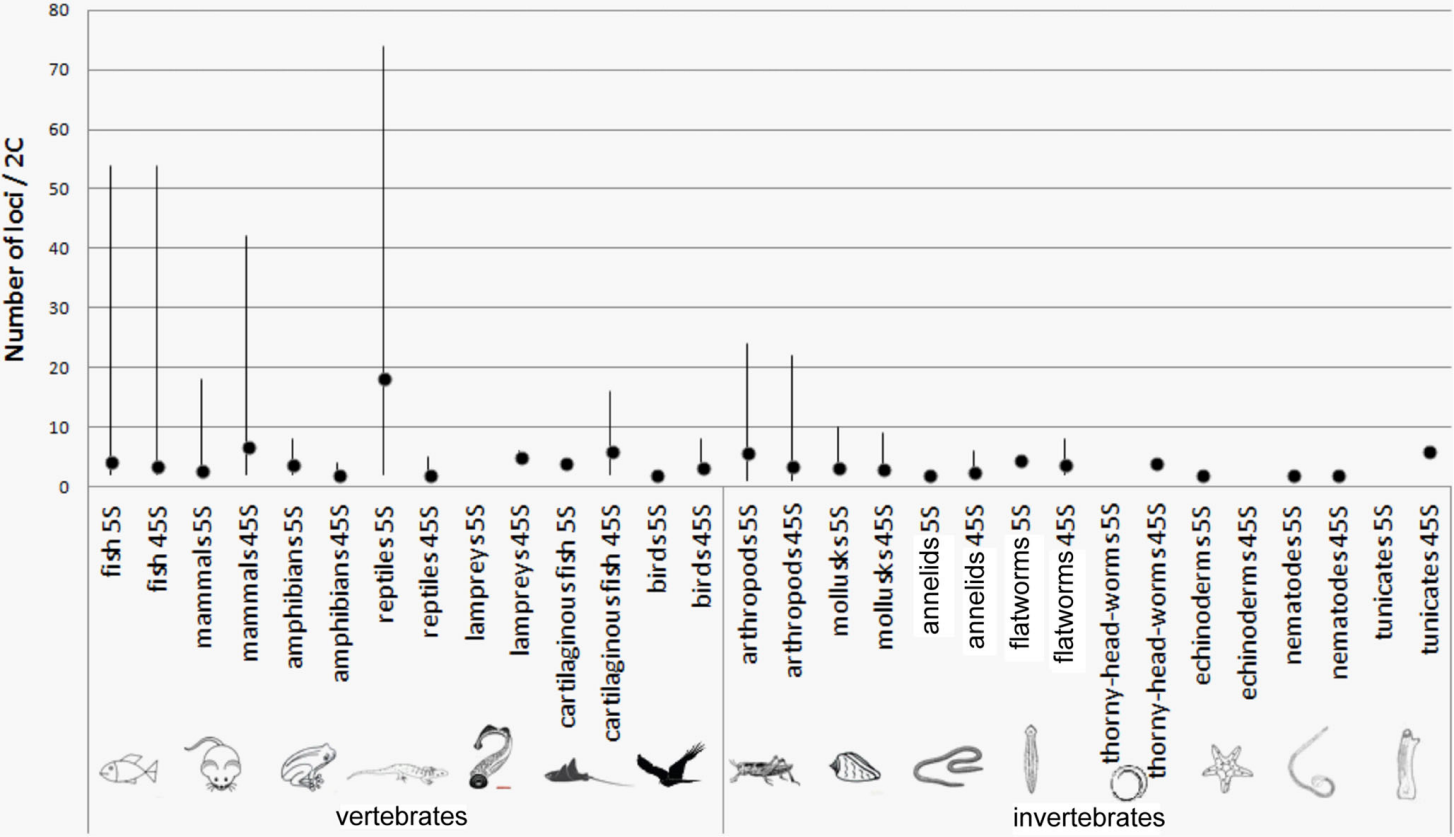
Number of 5S and 45S rDNA sites in different animal taxa. Values are presented for the diploid karyotypes. Black dots indicate the average number of sites per group; lines show the range. The relatively high average number of 5S sites in reptiles is explained by an exceptionally high number of loci recorded in some members of the Teiidae family (Carvalho et al. 2015) and generally few data available for this group

Considering the whole database (karyotypes), the average number of 45S and 5S sites per diploid chromosome set (2C) was 3.8 and 4.5, respectively (Supplementary Table S1). The median was two sites (single locus/1C) for both 45S and 5S rDNA, respectively, indicating that most karyotypes tend to maintain locus numbers moderately low. Relatively large differences between means and medians indicated a non-Gaussian distribution of values (also revealed by significant results in the Shapiro-Wilk tests). Indeed, in each group, we identified several karyotypes largely deviating from the average (Fig. 2). The maximum numbers of 45S sites were found in the Amazonian fish *Schizodon fasciatus* (54/2C, de Barros et al. 2017) and the brook trout *Salvelinus fontinalis* (50/2C, Fujiwara et al. 1998). In mammals, the maximum number of 45S loci was identified in *Mus pahari* (rodent) having 42 sites/2C (Britton-Davidian et al. 2012). The maximum numbers of 5S sites were found in the neotropical lizards from the Teiidae family, *Kentropyx calcarata* (68/2C) and *K. pelviceps* (74/2C) (Carvalho et al. 2015). These karyotypes apparently account for relatively high average number of 5S loci in reptiles (Fig. 2). In mammals, the highest number of 5S loci was found in *Rhinolophus hipposideros* (bat) having 18 sites/2C (Puerma et al. 2008). About 12% species showed variation at the species level (Supplementary Table S2). The variation is explained by the presence of rDNA loci in sex chromosomes and supernumerary B chromosomes (both particularly frequent in insects), polyploidy (mainly in fish), and overall interpopulation variation. One also has to consider variation arising from differential experimental approaches used in labs.

### Factors influencing rDNA loci multiplicity

About 60% karyotypes (766/1277) had a single 45S locus and 57% of karyotypes (358/628) had a single 5S locus. Karyotypes with multiple loci (both 5S and 45S) occurred almost in every group. In insects, multiple loci were found mostly in Orthoptera (e.g. grasshoppers, crickets, and locusts). These species are known to have relatively large genomes (Gregory 2017). Because of the known correlation between genome size and number of rDNA copies (Prokopowich et al. 2003), it is possible that dispersion of rDNA across chromosomes is related to their large genome sizes (~ 10 pg/2C, (Rees et al. 1978)). However, genome size cannot explain the high number of rDNA loci in actinopterygian fishes (e.g. Ráb et al. 2002; Mantovani et al. 2005; Cioffi et al. 2010; da Silva et al. 2011; Lima-Filho et al. 2014; Sember et al. 2015; Symonová et al. 2017) that generally harbour small genomes (~ 1 pg/2C). The increased number of loci could also be related to the large number of rDNA pseudogenes reported in some grasshopper genomes (Keller et al. 2006). In contrast, a whole genomic study in *Esox lucius* (Northern pike, fish) did not reveal increased pseudogenisation of highly (> 20,000 copies) amplified 5S genes (Symonová et al. 2017), suggesting that amplification does not automatically lead to pseudogenisation and that retention of pseudogenes varies between the genomes.

**Fig. 3:**
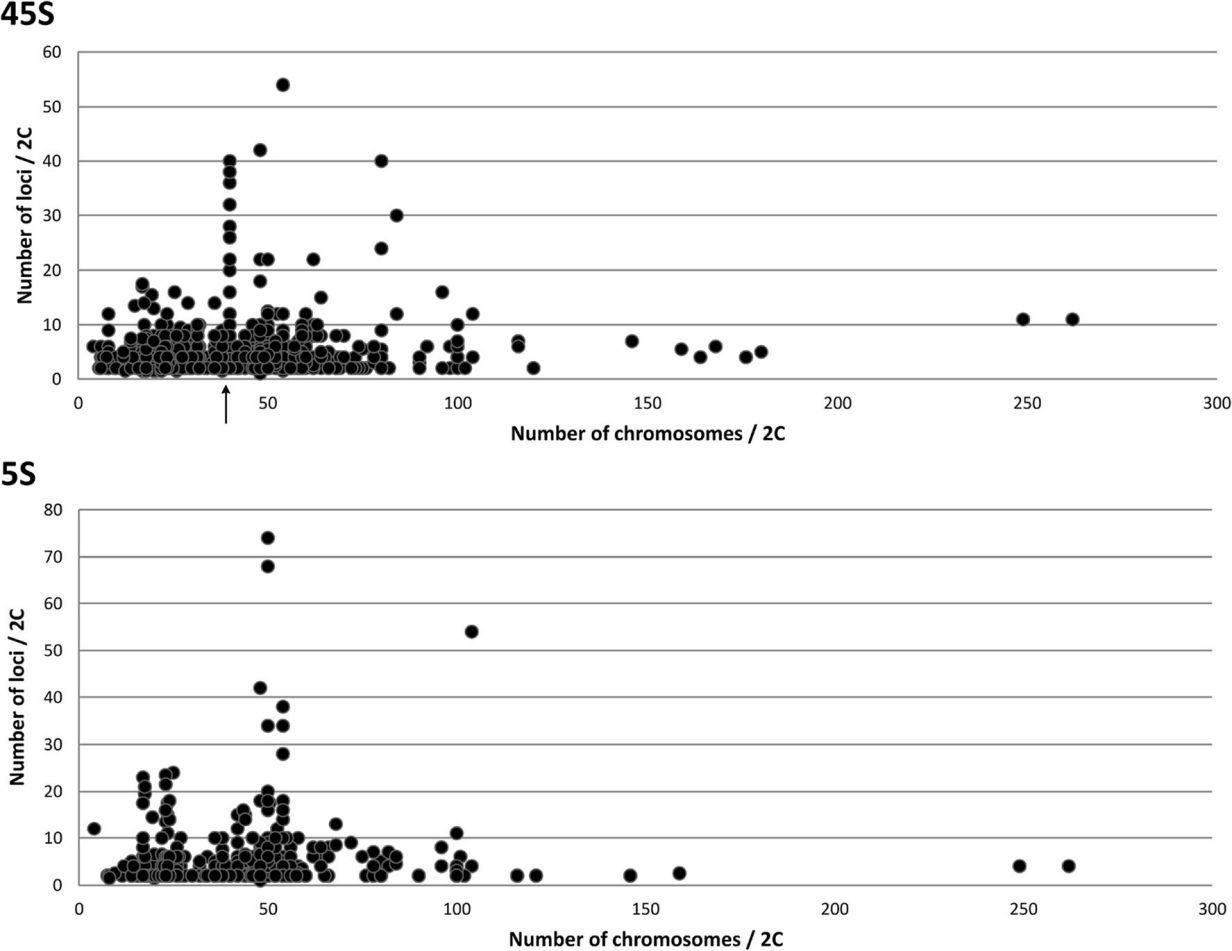
Plots showing a relationship between chromosome number (*x*-axis) and rDNA sites (*y*-axis). The arrow marks a typical 2n = 40 karyotype in the *Mus* genus showing the striking variation in the number of 45S but not 5S rDNA sites

The amplification of rDNA has often been attributed to polyploidy (Gornung 2013). However, species with extremely large number of chromosomes (> 100/2C) do not automatically exhibit a high number of loci (Fig. 3 and Supplementary Table S3). For example, members of the arthropod genus *Austropotamobius* (2n = 176) harbour only four 45S sites (Mlinarec et al. 2016). Similarly, the fish *Acipenser baerii* and *A. transmontanus* (2n = 262) show only moderate numbers of 5S (four) and 45S (11) sites (Fontana et al. 2003). Reduction of rDNA in these enlarged karyotypes could be related to the “genomic shock” following polyploidy events (Semon and Wolfe 2007; Garcia et al. 2017). On the other hand, some moderate karyotypes harbour high number of rDNA loci. For example, in *Ctenogobius smaragdus* (emerald goby, 2n = 48, Lima-Filho et al. 2014), *S. fontinalis* (fish, 2n = 84, Fujiwara et al. 1998), and *M. pahari* (mouse, 2n = 48, Cazaux et al. 2011), the loci were distributed across 91, 88, and 50% of chromosomes, respectively. Certainly, polyploidy cannot explain large numbers of loci in these species and other mechanisms such as interlocus recombination (Cazaux et al. 2011), transposon activity (Symonová et al. 2013), and integration of extrachromosomally replicated rDNA (Cohen et al. 2010) should be considered.

### Mutual relationships between 5S and 45S rDNA

Numerous case studies indicate likely independent amplification events of 5S and 45S rDNA in the genomes. For example, in the *Mus* genus, large (> 10-fold) variation occurs in 45S locus numbers (Britton-Davidian et al. 2012) without concomitant variation in 5S loci (Matsuda et al. 1994) (Fig. 3). Furthermore, two different cytotypes (2n = 52 and 2n = 54) of the Amazonian fish *Erythrinus erythrinus* (Cioffi et al. 2010) varied as much as 11-fold in the number of 5S loci while that of 45S loci was constant. Similarly, grasshopper genomes show extensive but independent variation in the number of 5S and 45S rDNA clusters (Cabral-de-Mello et al. 2011). Our comparative analysis of more than 500 karyotypes (Supplementary Fig. S1 and Supplementary Table S4) revealed that the numbers of both loci are not correlated (Pearson, *r* = 0.047, *p* value > 0.05). On the other hand, 43% karyotypes showed the same number of 45S and 5S loci, suggesting a potential relationship. However, the majority (89%) of equinumber karyotypes harboured a single locus of each, and the equinumber karyotypes with multiple loci were relatively rare (11%) which can be explained by a general tendency of genomes to keep the number of both loci low (Fig. 2).

In plants, equality of 45S and 5S loci was detected in 33% of karyotypes and a significant correlation (*p* < 0.005) between the number of 5S and 45S was observed (Garcia et al. 2017). This can be accounted to frequent whole genome duplications in plants through which both loci are equally multiplied. In animals, about 75% of karyotypes had 5S and 45S loci on different chromosomes (separate arrangement), while 25% of karyotypes had at least one chromosome bearing both loci (colocalised). Thus, a tendency towards 5S and 45S colocalisation on the same chromosome does not appear to be as strong as in plants where colocalisation occurs in 58% of genera (Roa and Guerra 2015). Perhaps, this could be related to the increased number of loci in plants (median for 45S and 5S sites is 4/2C) (Roa and Guerra 2012; Garcia et al. 2017)) compared to the animals where medians are generally lower (2 sites/2C (Supplementary Table S1)). Colocalisation may also stimulate recombination frequency between both loci possibly leading to their physical linkage and formation of 45S-5S units. Of note, linked 45S-5S units are relatively common in plants (Garcia et al. 2009; Wicke et al. 2011; Garcia and Kovarik 2013) while in animals, they have been described in few arthropods (Drouin et al. 1992) and crustaceans (Drouin and de Sá 1995) so far. The number of 5S and 45S rRNA gene copies seems to be harmonised following concerted copy number variation in human and mouse (Gibbons et al. 2015). Thus, there may not be a simple relationship between the number of loci and the number of copies since gene richness may differ between loci. In this context, the size of nucleoli has been correlated with the number of 45S ribosomal RNA genes in amphibians (Miller and Brown 1969).

### Position of rDNA on chromosomes

**Fig. 4:**
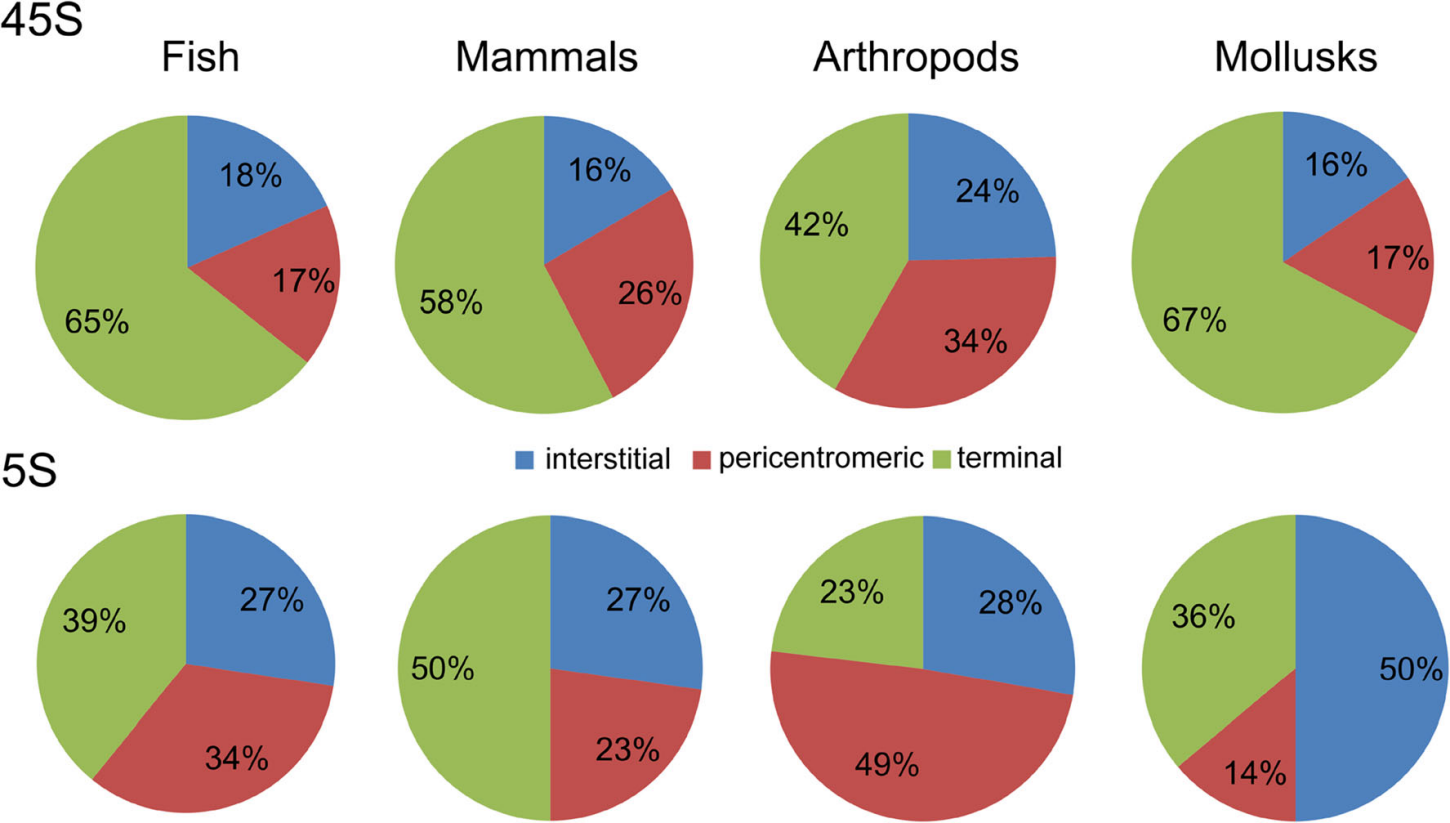
Position of rDNA sites in chromosomes. The numbers of 45S and 5S sites counted in each group are as follows: fish (*N* = 479 and *N* = 417, respectively), mammals (*N* = 156 and *N* = 40, respectively), arthropods (*N* = 424 and *N* = 96, respectively), and mollusks (*N* = 54 and *N* = 33, respectively). The source data are given in Supplementary Table S5

**Fig. 5:**
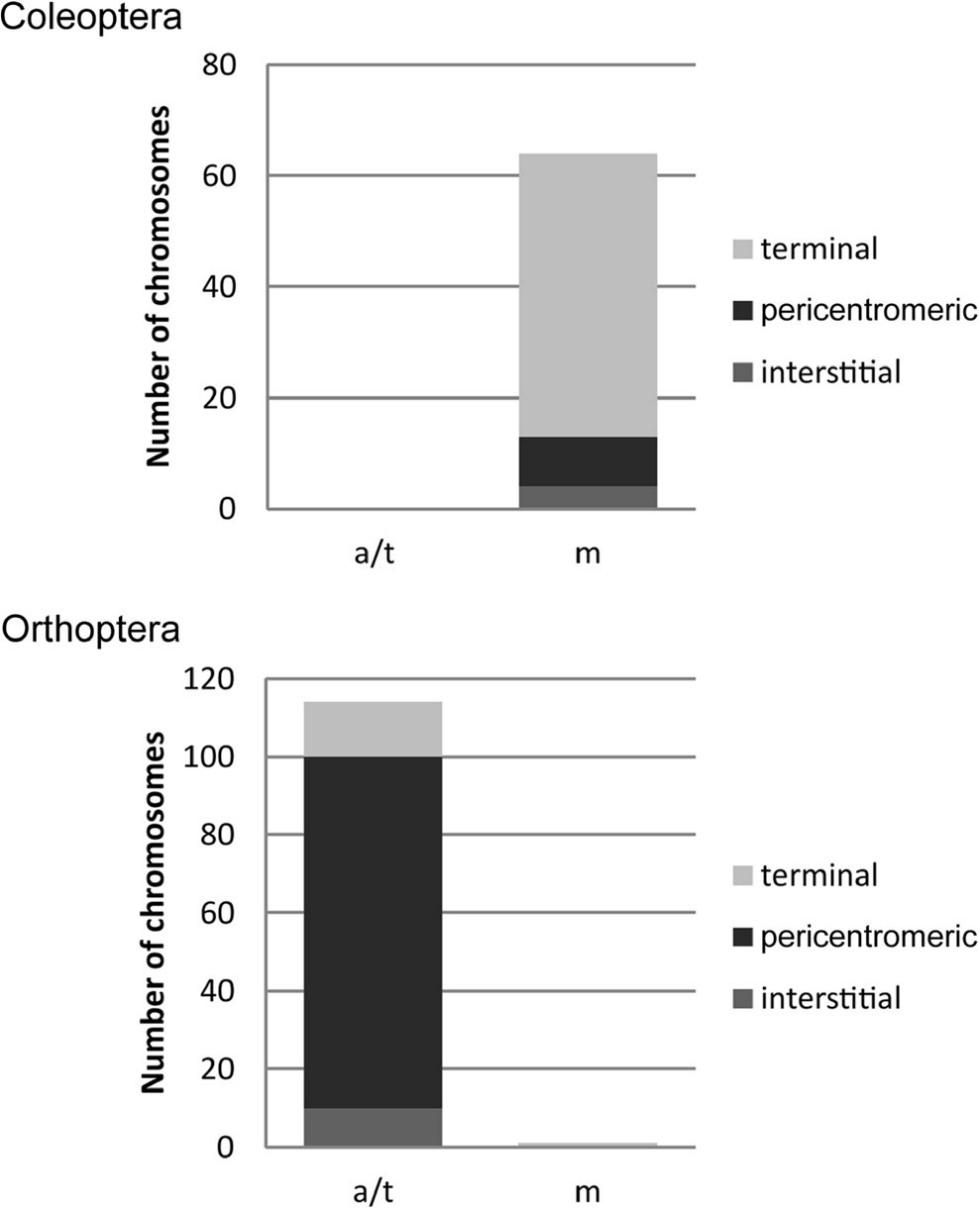
Relationship between chromosome morphology (x-axis) and 45S rDNA positions (y-axis) in two of the largest orders of insects, Coleoptera (*N* = 85) and Orthoptera (*N* = 141). Only chromosomes with well-resolved morphologies were considered for the analysis. Chromosome type: m—metacentric/submetacentric; a/t—acrocentric/telocentric. The source datasets are in Supplementary Table S7

In the literature, there have been considerable debates over “randomness” of rDNA chromosomal positions (Hillis and Dixon 1991; Gornung 2013; Roa and Guerra 2015; Garcia et al. 2017). The information gathered in this database allowed us to address the question of preferential position (if any) of rDNA in chromosomes. We selected groups (Fig. 4) containing at least 40 species allowing robust statistical evaluation. The pie charts (Fig. 4 and Supplementary Table S5) show distribution of loci along different parts of chromosomes. Although it is clear that rDNA may occur at nearly any chromosomal position, there were significant trends in particular groups of animals. A distal location of 45S is clearly preferred in mammals, fish, and mollusks while in arthropods, its distribution is more balanced. The 5S loci were more evenly placed along the chromosomes than the 45S loci, consistent with previous observations (Baumlein and Wobus 1976; Roa and Guerra 2015; Garcia et al. 2017). In arthropods, the proximal positions of rDNA loci (both 5S and 45S) were significantly more common than in other groups (Fig. 4, for a statistical support, see Supplementary Table S6). Arthropods are the largest and most diversified phylum, representing around 70% of all animals (IUCN 2014). Since insects are highly represented within arthropods and also in our database (Table 1), we analysed 45S rDNA positions in its two largest orders, Coleoptera (beetles) and Orthoptera (mostly grasshoppers and crickets). Strikingly, significant (Supplementary Table S7) differences in 45S rDNA positions were found between both groups: Coleoptera had mostly distal distribution of 45S loci while Orthoptera had these genes preferentially located at pericentromeric positions (Fig. 5), and terminal positions were found only exceptionally (Veltsos et al. 2009).

There are several caveats in determining rDNA position of chromosomes. First, many karyotypes harbour chromosomes that are too small, preventing the accurate determination of loci positions. This is particularly the case of species with high number of chromosomes and relatively small genomes. Second, the resolution of FISH experiments may not permit to ascertain whether a site is located closer to the centromere or to the telomere in telocentric chromosomes or in short arms of acrocentric chromosomes. In these morphological types, the rDNA position could be considered either distal (as it appears at the end of the chromosome) or proximal (as it is located in the terminal centromere characterising these chromosomes). Hence, the “proximal-distal” location could be a more appropriate term for “pericentromeric” rDNA in acrocentric and telocentric chromosomes. For such reasons, the information about the position on chromosomes should be taken with great care since interpretation of FISH signals may vary between the researchers.

### Are there functional constrains for the maintenance of distinct rDNA positions?

More than 50% of karyotypes in the database had 45S rDNA at distal (subtelomeric) positions. The number of sites located close to the chromosome ends could actually be even higher since many proximal locations can be considered as proximal-distal (78%, Supplementary Table S8). The question arises as to the functional significance (if any) of these observations, made independently in both animals and plants (Lima-de-Faria 1976; Roa and Guerra 2012; Garcia et al. 2017):

I. Position of 45S rDNA close to chromosome ends may be important for accurate positioning of 45S rDNA chromatin within and around the nucleolus (Gornung 2013). It is known that during mitosis, parts of the nucleolar proteins remain at the NORs (Schwarzacher and Wachtler 1993). Perhaps, association of partially decondensed rDNA chromatin with these proteins is better maintained at distal (or “distal-proximal”) than at interstitial or centromeric positions during the transfer through mitosis. If so, distally positioned loci may better secure that rDNA transcription is rapidly resumed following mitosis early after cell division, perhaps via specific chromatin configuration. However, pericentromeric NORs were found in metacentric chromosomes of several single locus karyotypes (e.g. Barth et al. 2013; Singh and Barman 2013) suggesting that these positions, although infrequent (2% karyotypes; Supplementary Table S8), are probably compatible with expression of residing rDNA. Furthermore, secondary constrictions, thought to be remnants of activity in previous interphase, were identified at interstitial positions in some species (Fagundes et al. 2003). These studies suggest that there are no functional constrains limiting position of NOR in chromosomes with respect to the nuclear topology.
II. Non-coding functions of 45S rDNA should be considered (Kobayashi 2008). Perhaps, rDNA heterochromatin could fulfil a structural function contributing to the stabilisation of telomeric and centromeric (in distal-proximal positions) domains. In this regard, the principal determinant in rDNA silencing, the nucleolar remodelling complex (NoRC), is also important to maintain genome stability (Guetg et al. 2010) and the formation of heterochromatin (Postepska-Igielska and Grummt 2014). Cazaux et al. (2011) proposed that rDNA may predispose the chromatin to centromere formation. Indeed, pseudogenised rDNA copies that seem to regularly occur in different genomes at variable frequencies (Mentewab et al. 2011; Wang et al. 2016; Robicheau et al. 2017) may homogenise and even evolve in independent satellites (Lim et al. 2004; Ferreira et al. 2007).
III. Concerted evolution may be more efficient at chromosome termini than in other regions. It is well established that rDNA evolves via the “concerted evolution” model that maintains homogeneity of multigenic families (Zimmer et al. 1981; Dover 1982). Gene conversion and non-homologous recombination are the major players of concerted evolution (reviewed in Nieto Feliner and Rosselló 2012). The regions near the ends of chromosomes of several organisms show higher recombination rates than more centric sequences (McKim et al. 1988; Jensen-Seaman et al. 2004). Functional rDNA copies may be located in chromosome sites with intensive recombination in subtelomeric regions and hence these positions would be favoured by natural selection. Yet, patterns of 45S rDNA unit divergence seem to be similar in species with distal (humans, Gonzalez and Sylvester 2001) and proximal locations (house mouse, Sasaki et al. 1987). However, in the *Mus* genus, 45S rDNA loci are preferentially positioned at telocentric chromosome close to the chromosome ends, which better correspond to proximal-distal location defined above. Cazaux et al. (2011) proposed that a specific configuration of these specific domains in interphase may stimulate meiotic recombination between non homologous loci.

## Electronic supplementary material

Supplementary Fig. S1 Relationship between the number of 45S and 5S rDNA sites. Note, species with extremely high (> 10/2C) number of 5S loci do not usually show extremely high number of 45S loci and vice versa. The source data are given in Supplementary Table S4. (GIF 18 kb)

High resolution image (TIFF 448 kb)

Supplementary Table S1 (PDF 364 kb)

Supplementary Table S2 (PDF 278 kb)

Supplementary Table S3 (PDF 699 kb)

Supplementary Table S4 (PDF 461 kb)

Supplementary Table S5 (PDF 443 kb)

Supplementary Table S6 (PDF 447 kb)

Supplementary Table S7 (PDF 438 kb)

Supplementary Table S8 (PDF 594 kb)

## References

[CR1] Averbeck KT, Eickbush TH (2005). Monitoring the mode and tempo of concerted evolution in the *Drosophila melanogaster* rDNA locus. Genetics.

[CR2] Barth A, Souza VA, Sole M, Costa MA (2013). Molecular cytogenetics of nucleolar organizer regions in *Phyllomedusa* and *Phasmahyla* species (Hylidae, Phyllomedusinae): a cytotaxonomic contribution. Geneti Mol Res.

[CR3] Baumlein H, Wobus U (1976). Chromosomal localization of ribosomal 5S RNA genes in *Chironomus thumni* by in situ hybridization of iodinated 5S RNA. Chromosoma.

[CR4] Britton-Davidian J, Cazaux B, Catalan J (2012). Chromosomal dynamics of nucleolar organizer regions (NORs) in the house mouse: micro-evolutionary insights. Heredity.

[CR5] Cabral-de-Mello DC, Oliveira SG, de Moura RC, Martins C (2011). Chromosomal organization of the 18S and 5S rRNAs and histone H3 genes in *Scarabaeinae coleopterans*: insights into the evolutionary dynamics of multigene families and heterochromatin. BMC Genet.

[CR6] Cabrero J, Perfectti F, Gomez R, Camacho JPM, Lopez-Leon MD (2003) Population variation in the A chromosome distribution of satellite DNA and ribosomal DNA in the grasshopper *Eyprepocnemis plorans*. Chromosom Res 11:375–381, 4, DOI: 10.1023/A:1024127525756 10.1023/a:1024127525756 12906134

[CR7] Carvalho NDM, Pinheiro VSS, Carmo EJ, Goll LG, Schneider CH, Gross MC (2015). The organization of repetitive DNA in the genomes of Amazonian lizard species in the family Teiidae. Cytogenet Genome Res.

[CR8] Castro J, Rodriguez S, Pardo BG, Sanchez L, Martinez P (2001) Population analysis of an unusual NOR-site polymorphism in brown trout (*Salmo trutta* L.) Heredity (3):291–286, 302. 10.1046/j.1365-2540.2001.00834.x 10.1046/j.1365-2540.2001.00834.x 11488966

[CR9] Cazaux B, Catalan J, Veyrunes F, Douzery EJP, Britton-Davidian J (2011). Are ribosomal DNA clusters rearrangement hotspots? A case study in the genus *Mus* (Rodentia, Muridae). BMC Evol Biol.

[CR10] Cioffi MB, Martins C, Bertollo LAC (2010). Chromosome spreading of associated transposable elements and ribosomal DNA in the fish *Erythrinus erythrinus*. Implications for genome change and karyoevolution in fish. BMC Evol Biol.

[CR11] Cohen S, Agmon N, Sobol O, Segal D (2010). Extrachromosomal circles of satellite repeats and 5S ribosomal DNA in human cells. Mob DNA.

[CR12] da Silva M, Matoso DA, Vicari MR, de Almeida MC, Margarido VP, Artoni RF (2011). Physical mapping of 5S rDNA in two species of knifefishes: *Gymnotus pantanal* and *Gymnotus paraguensis* (Gymnotiformes). Cytogenet Genome Res.

[CR13] de Barros LC, Galetti PM, Feldberg E (2017). Mapping 45S and 5S ribosomal genes in chromosomes of Anostomidae fish species (Ostariophysi, Characiformes) from different Amazonian water types. Hydrobiologia.

[CR14] Dover GA (1982). Molecular drive: a cohesive mode of species evolution. Nature.

[CR15] Drouin G, de Sá MM (1995). The concerted evolution of 5S ribosomal genes linked to the repeat units of other multigene families. Mol Biol Evol.

[CR16] Drouin G, Sevigny JM, McLaren IA, Hofman JD, Doolittle WF (1992). Variable arrangement of 5S ribosomal genes within the ribosomal DNA repeats of arthropods. Mol Biol Evol.

[CR17] Dubcovsky J, Dvorak J (1995). Ribosomal RNA multigene loci—nomads of the Triticeae genomes. Genetics.

[CR18] Fagundes V, Christoff AU, Amaro-Ghilard RC, Scheibler DR, Yonenaga-Yassuda Y (2003). Multiple interstitial ribosomal sites (NORs) in the Brazilian squirrel *Sciurus aestuans ingrami* (Rodentia, Sciuridae) with 2n = 40. An overview of *Sciurus cytogenetics*. Genet Mol Biol.

[CR19] Ferreira IA, Bertollo LAC, Martins C (2007). Comparative chromosome mapping of 5S rDNA and 5SHindIII repetitive sequences in Erythrinidae fishes (Characiformes) with emphasis on the *Hoplias malabaricus* ‘species complex’. Cytogenet Genome Res.

[CR20] Fontana F, Lanfredi M, Congiu L, Leis M, Chicca M, Rossi R (2003). Chromosomal mapping of 18S-28S and 5S rRNA genes by two-colour fluorescent *in situ* hybridization in six sturgeon species. Genome.

[CR21] Fujiwara A, Abe S, Yamaha E, Yamazaki F, Yoshida MC (1998). Chromosomal localization and heterochromatin association of ribosomal RNA gene loci and silver-stained nucleolar organizer regions in salmonid fishes. Chromosom Res.

[CR22] Garcia S, Garnatje T, Kovařík A (2012). Plant rDNA database: ribosomal DNA *loci* data including other karyological and cytogenetic information in plants. Chromosoma.

[CR23] Garcia S, Kovarik A (2013). Dancing together and separate again: gymnosperms exhibit frequent changes of fundamental 5S and 35S rRNA genes (rDNA) organisation. Heredity.

[CR24] Garcia S, Kovařík A, Leitch AR, Garnatje T (2017). Cytogenetic features of rRNA genes across land plants: analysis of the plant rDNA database. Plant J.

[CR25] Garcia S, Lim KY, Chester M, Garnatje T, Pellicer J, Valles J, Leitch AR, Kovařík A (2009). Linkage of 35S and 5S rRNA genes in *Artemisia* (family Asteraceae): first evidence from angiosperms. Chromosoma.

[CR26] Gibbons JG, Branco AT, Godinho SA, Yu S, Lemos B (2015). Concerted copy number variation balances ribosomal DNA dosage in human and mouse genomes. Proc Natl Acad Sci U S A.

[CR27] Gonzalez IL, Sylvester JE (2001). Human rDNA: evolutionary patterns within the genes and tandem arrays derived from multiple chromosomes. Genomics.

[CR28] Gornung E (2013). Twenty years of physical mapping of major ribosomal RNA genes across the teleosts: a review of research. Cytogenet Genome Res.

[CR29] Gregory TR (2017) Animal genome size database. http://www.genomesize.com/. Accessed 26 January 2017

[CR30] Guetg C, Lienemann P, Sirri V, Grummt I, Hernandez-Verdun D, Hottiger MO, Fussenegger M, Santoro R (2010). The NoRC complex mediates the heterochromatin formation and stability of silent rRNA genes and centromeric repeats. EMBO J.

[CR31] Hillis DM, Dixon MT (1991). Ribosomal DNA—molecular evolution and phylogenetic inference. Q Rev Biol.

[CR32] IUCN (2014) Red list of threatened species. Version 2014.3. Summary statistics for globally threatened species. Table 1: numbers of threatened species by major groups of organisms (1996–2014), www.iucnredlist.org. Accessed 20 January 2017

[CR33] Jensen-Seaman MI, Furey TS, Payseur BA, Lu Y, Roskin KM, Chen CF, Thomas MA, Haussler D, Jacob HJ (2004). Comparative recombination rates in the rat, mouse, and human genomes. Genome Res.

[CR34] Keller I, Chintauan-Marquier IC, Veltsos P, Nichols RA (2006). Ribosomal DNA in the grasshopper *Podisma pedestris*: escape from concerted evolution. Genetics.

[CR35] Kobayashi T (2008). A new role of the rDNA and nucleolus in the nucleus-rDNA instability maintains genome integrity. BioEssays.

[CR36] Leitch AR, Schwarzacher T, Jackson D, Leitch IJ (1994). *In situ* hybridization: a practical guide.

[CR37] Lim KY, Skalická K, Koukalová B, Volkov RA, Matyasek R, Hemleben V, Leitch AR, Kovařík A (2004). Dynamic changes in the distribution of a satellite homologous to intergenic 26-18S rDNA spacer in the evolution of *Nicotiana*. Genetics.

[CR38] Lima-de-Faria A (1976). The chromosome field I. Prediction of the location of ribosomal citrons. Hereditas.

[CR39] Lima-Filho PA, Bertollo LA, Cioffi MB, Costa GW, Molina WF (2014). Karyotype divergence and spreading of 5S rDNA sequences between genomes of two species: darter and emerald gobies (Ctenogobius, Gobiidae). Cytogenet Genome Res.

[CR40] Lohe AR, Roberts PA (1990). An unusual Y chromosome of *Drosophila simulans* carrying amplified rDNA spacer without rRNA genes. Genetics.

[CR41] Mantovani M, Abel LD, Moreira-Filho O (2005). Conserved 5S and variable 45S rDNA chromosomal localisation revealed by FISH in *Astyanax scabripinnis* (Pisces, Characidae). Genetica.

[CR42] Matsuda Y, Moriwaki K, Chapman VM, Hoi-Sen Y, Akbarzadeh J, Suzuki H (1994). Chromosomal mapping of mouse 5S rRNA genes by direct R-banding fluorescence in situ hybridization. Cytogenet Cell Genet.

[CR43] McKim KS, Howell AM, Rose AM (1988). The effects of translocations on recombination frequency in *Caenorhabditis elegans*. Genetics.

[CR44] McTaggart S, Dudycha JL, Omilian A, Crease TJ (2007). Rates of recombination in the ribosomal DNA of apomictically propagated *Daphnia obtusa* lines. Genetics.

[CR45] Mentewab AB, Jacobsen MJ, Flowers RA (2011). Incomplete homogenization of 18 S ribosomal DNA coding regions in *Arabidopsis thaliana*. BMC Res Notes.

[CR46] Miller L, Brown DD (1969). Variation in the activity of nucleolar organizers and their ribosomal gene content. Chromosoma.

[CR47] Mlinarec J, Porupski I, Maguire I, Klobucar G (2016). Comparative karyotype investigations in the white-clawed crayfish *Austropotamobius pallipes* (Lereboullet, 1858) species complex and stone crayfish *A. torrentium* (Schrank, 1803) (Decapoda: Astacidae). J Crust. Biol.

[CR48] Nieto Feliner G, Rosselló JA, Wendel JF (2012). Concerted evolution of multigene families and homeologous recombination. Plant Genome Diversity.

[CR49] Pinkel D, Straume T, Gray JW (1986). Cytogenetic analysis using quantitative, high-sensitivity, fluorescence hybridization. Proc Natl Acad Sci U S A.

[CR50] Postepska-Igielska A, Grummt I (2014). NoRC silences rRNA genes, telomeres, and centromeres. Cell Cycle.

[CR51] Prokopowich CD, Gregory TR, Crease TJ (2003). The correlation between rDNA copy number and genome size in eukaryotes. Genome.

[CR52] Puerma E, Acosta MJ, Barragán MJ, Martínez S, Marchal JA, Bullejos M, Sánchez A (2008). The karyotype and 5S rRNA genes from Spanish individuals of the bat species *Rhinolophus hipposideros* (Rhinolophidae; Chiroptera). Genetica.

[CR53] Ráb P, Crossman EJ, Reed KM, Rábová M (2002) Chromosomal characteristics of ribosomal DNA in two extant species of North American mudminnows <i>Umbra pygmaea</i> and <i>U. limi</i> (Euteleostei: Umbridae). Cytogenet Genome Res 98(2-3):194–198 10.1159/000069800 12698003

[CR54] Rees H, Shaw DD, Wilkinson P (1978) Nuclear DNA Variation among Acridid Grasshoppers. Proc R Soc B Biol Sci 202(1149):517–525

[CR55] Roa F, Guerra M (2012) Distribution of 45S rDNA sites in chromosomes of plants: Structural and evolutionary implications. BMC Evol Biol 12(1):225 10.1186/1471-2148-12-225 PMC3583730 23181612

[CR56] Roa F, Guerra M (2015) Non-Random Distribution of 5S rDNA Sites and Its Association with 45S rDNA in Plant Chromosomes. Cytogenet Genome Res 146(3):243–249 10.1159/000440930 26489031

[CR57] Robicheau BM, Susko E, Harrigan AM, Snyder M (2017) Ribosomal RNA Genes Contribute to the Formation of Pseudogenes and Junk DNA in the Human Genome. Genome Biol Evol 9(2):380–397 10.1093/gbe/evw307 PMC5381670 28204512

[CR58] Roy V, Monti-Dedieu L, Chaminade N, Siljak-Yakovlev S, Aulard S, Lemeunier F, Montchamp- Moreau C (2005) Evolution of the chromosomal location of rDNA genes in two Drosophila species subgroups: ananassae and melanogaster. Heredity (Edinb) 94:388–395 10.1038/sj.hdy.6800612 15726113

[CR59] Schubert I, Wobus U (1985) In situ hybridization confirms jumping nucleolus organizing regions in Allium. Chromosoma 92(2):143–148

[CR60] Schwarzacher HG, Wachtler F (1993). The nucleolus. Anat Embryol (Berl).

[CR61] Sember A, Bohlen J, Slechtová V, Altmanová M, Symonová R, Rab P (2015) Karyotype differentiation in 19 species of river loach fishes (Nemacheilidae, Teleostei): extensive variability associated with rDNA and heterochromatin distribution and its phylogenetic and ecological interpretation. BMC Evol Biol 15:251 10.1186/s12862-015-0532-9 PMC4647339 26573692

[CR62] Singh M, Barman AS (2013) Chromosome breakages associated with 45S ribosomal DNA sequences in spotted snakehead fish Channa punctatus. Mol Biol Rep 40(1):723–729 10.1007/s11033-012-2112-z 23065230

[CR63] Symonová R, Majtanová Z, Sember A, Staaks GBO, Bohlen J, Freyhof J, Rabová M, Rab P (2013) Genome differentiation in a species pair of coregonine fishes: an extremelyrapid speciation driven by stress-activated retrotransposons mediating extensive ribosomal DNA multiplications. BMC Evol Biol 13:42 10.1186/1471-2148-13-42 PMC3585787 23410024

[CR64] Symonová R, Ocalewicz K, Kirtiklis L, Delmastro GB, Pelikánová Š, Garcia S, Kovařík A (2017) Higher-order organisation of extremely amplified, potentially functional and massively methylated 5S rDNA in European pikes (Esox sp.) BMC Genomics 18(1):391 10.1186/s12864-017-3774-7 PMC5437419 28521734

[CR65] Tanomtong A, Khunsook S, Keawmad P, Pintong K (2008) Cytogenetic Study of the Leopard, Panthera pardus (Carnivora, Felidae) by Conventional Staining, G-banding and High-resolution Staining Technique. Cytologia 73(1):81–90

[CR66] Veltsos P, Keller I, Nichols RA (2009) Geographically localised bursts of ribosomal DNA mobility in the grasshopper Podisma pedestris. Heredity 103(1):54–61 10.1038/hdy.2009.32 19384343

[CR67] Wang W, Lu M, Becher H, Garcia S, Kovarikova A, Leitch IJ, Leitch AR, Kovarik A (2016) Astonishing 35S rDNA diversity in the gymnosperm species Cycas revoluta Thunb. Chromosoma 125(4):683–699 10.1007/s00412-015-0556-3 PMC5023732 26637996

[CR68] Wang M, Lemos B, Eng C (2017) Ribosomal DNA copy number amplification and loss in human cancers is linked to tumor genetic context, nucleolus activity, and proliferation. PLoS Genet 13(9):e1006994 10.1371/journal.pgen.1006994 PMC5605086 28880866

[CR69] Wicke S, Costa A, Muñoz J, Quandt D (2011) Restless 5S: The re arrangement(s) and evolution of the nuclear ribosomal DNA in land plants. Mol Phylogenet Evol 61(2):321–332 10.1016/j.ympev.2011.06.023 21757016

[CR70] Zimmer EA, Martin SL, Beverley SM, Kan YW, Wilson AC (1981) The untranslated regions of beta-globin mRNA evolve at a functional rate in higher primates. Proc Natl Acad Sci U S A 77(4):2158–2162 10.1016/0092-8674(81)90181-1 7285116

