# Supplementary figures and images for: Evolutionary trends in animal ribosomal DNA loci: introduction to a new online database
Source: Chromosoma. 2017 Nov 30;127(1):141–50. doi: 10.1007/s00412-017-0651-8 (PMC5818627; doi:10.1007/s00412-017-0651-8)

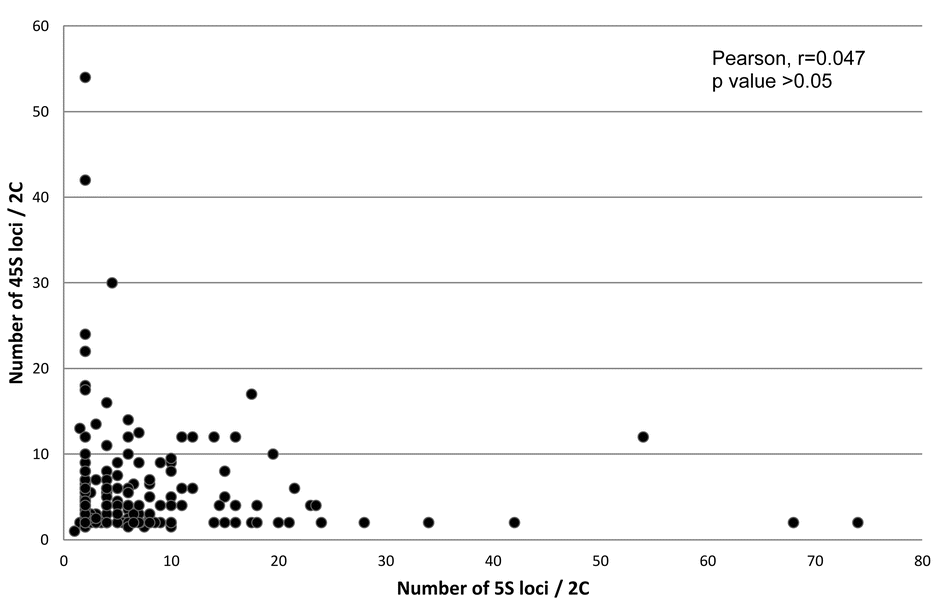

Supplement: Supplementary file 1 — Relationship between the number of 45S and 5S rDNA sites. Note, species with extremely high (> 10/2C) number of 5S loci do not usually show extremely high number of 45S loci and vice versa. The source data are given in Supplementary Table S4. (GIF 18 kb) [file 412_2017_651_Fig6_ESM.gif]

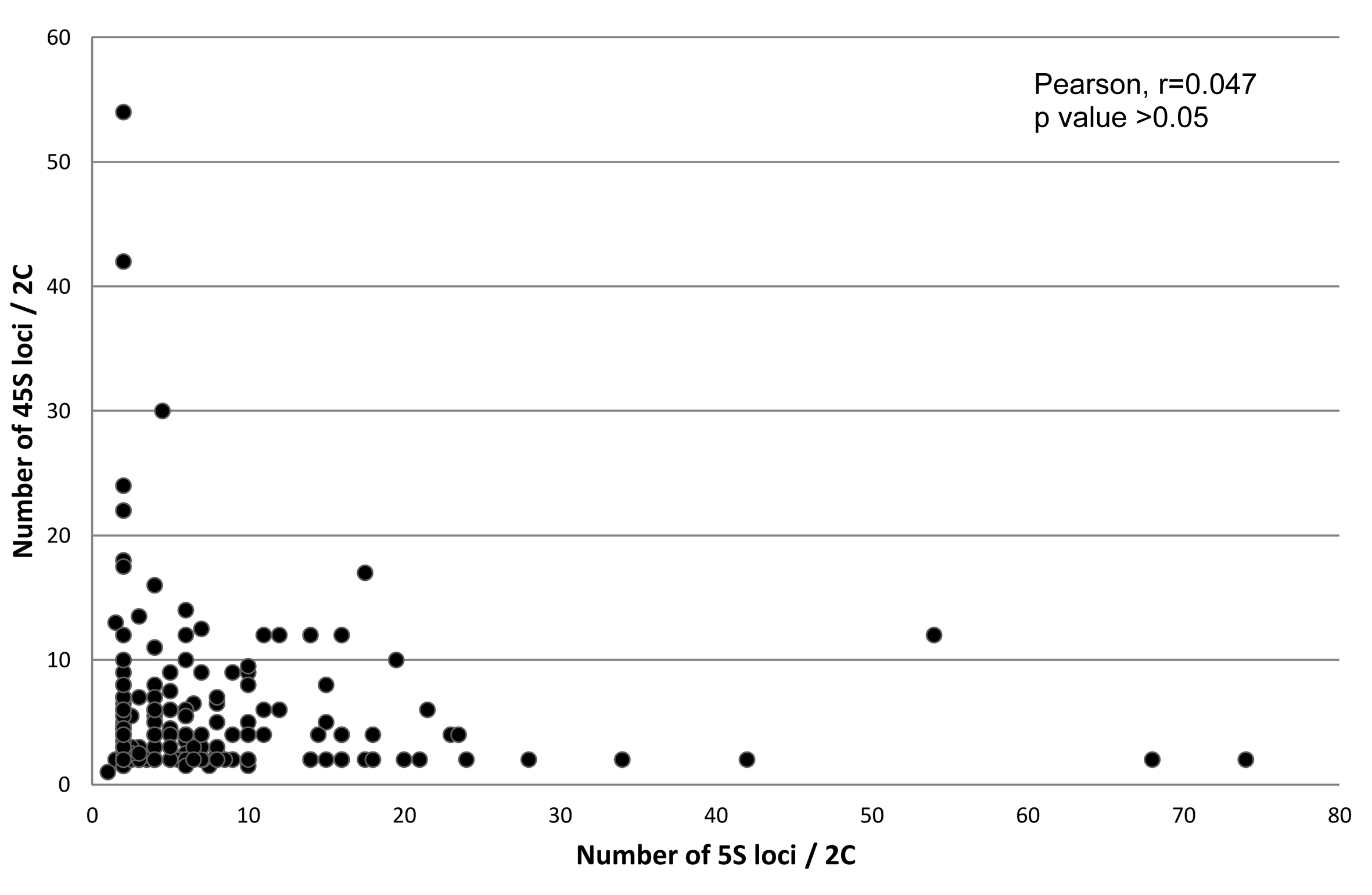

Supplement: Supplementary file 2 — High resolution image (TIFF 448 kb) [file 412_2017_651_MOESM1_ESM.tif]
