# Supplementary material for: Evolutionary trends in animal ribosomal DNA loci: introduction to a new online database
Source: Chromosoma. 2017 Nov 30;127(1):141–50. doi: 10.1007/s00412-017-0651-8 (PMC5818627; doi:10.1007/s00412-017-0651-8)
Supplement: Supplementary file 3 — (PDF 364 kb) [file 412_2017_651_MOESM2_ESM.pdf]

Supplementary Table S1. Range, average and median of rDNA sites in different groups of animals

Title: Evolutionary trends in animal ribosomal DNA loci: introduction to a new online database

Authors: Jana Sochorová[1*], Sònia Garcia[2*], Francisco Gálvez[3], Radka Symonová[4], Aleš Kovařík[1§]

Address: [1]*Institute of Biophysics, Academy of Sciences of the Czech Republic, Brno CZ–61265, Czech Republic.*

[2] *Institut Botànic de Barcelona (IBB-CSIC-ICUB), Passeig del Migdia s/n, 08038 Barcelona, Catalonia, Spain.*

[3]*Bioscripts - Centro de Investigación y Desarrollo de Recursos Científicos, 41012 Sevilla, Andalusia, Spain.*

[4]*Faculty of Science, University of Hradec Kralove, Hradecka 1285, Hradec Kralove CZ-50003, Czech Republic*

5S rDNA	Min	Max	Average	Median
**All kayotypes**	**1**	**74**	**4.51**	**2**
**Total vertebrates**	**2**	**74**	**4.37**	**2**
**Total invertebrates**	**1**	**24**	**5.03**	**2**
fish	2	54	4.21	2
mammals	2	18	2.83	2
amphibians	2	8	3.69	2
reptiles	2	74	18.18	4
cartilaginous fish	4	4	4.00	4
birds	2	2	2.00	2
arthropods	1	24	5.81	4
mollusks	2	10	3.18	2
annelids	2	2	2.00	n.d.
flatworms	4	5	4.50	4.5
echinoderms	2	2	n.d.	n.d.
nematodes	2	2	n.d.	n.d.

45S rDNA	Min	Max	Average	Median
**All kayotypes**	**1**	**54**	**3.79**	**2**
**Total vertebrates**	**1**	**54**	**3.99**	**2**
**Total invertebrates**	**2**	**22**	**3.49**	**2**
fish	2	54	3.48	2
mammals	2	42	6.87	6
amphibians	1	4	2.10	2.5
reptiles	1	5	2.13	2
lampreys	4	6	5.00	4
cartilaginous fish	2	16	6.00	2
birds	2	8	3.20	2
arthropods	1	22	3.58	2
mollusks	2	9	2.94	2
annelids	2	6	2.55	2
flatworms	2	8	3.81	2
thorny-head-worms	4	4	4.00	4
nematodes	2	2	n.d.	n.d.
tunicates	6	6	n.d.	n.d.
