# Supplementary material for: Evolutionary trends in animal ribosomal DNA loci: introduction to a new online database
Source: Chromosoma. 2017 Nov 30;127(1):141–50. doi: 10.1007/s00412-017-0651-8 (PMC5818627; doi:10.1007/s00412-017-0651-8)
Supplement: Supplementary file 4 — (PDF 278 kb) [file 412_2017_651_MOESM3_ESM.pdf]

Supplementary Table S2. Intraspecific variability in locus numbers

Title: Evolutionary trends in animal ribosomal DNA loci: introduction to a new online database
Authors: Jana Sochorová[1*], Sònia Garcia[2*], Francisco Gálvez[3], Radka Symonová [4], Aleš Kovařík[1§]
Address: [1]*Institute of Biophysics, Academy of Sciences of the Czech Republic, Brno CZ–61265, Czech Republic.*
[2] *Institut Botànic de Barcelona (IBB-CSIC-ICUB), Passeig del Migdia s/n, 08038 Barcelona, Catalonia, Spain.*
[3]*Bioscripts - Centro de Investigación y Desarrollo de Recursos Científicos, 41012 Sevilla, Andalusia, Spain.*
[4]*Faculty of Science, University of Hradec Kralove, Hradecka 1285, Hradec Kralove CZ-50003, Czech Republic*

5S rDNA

	Number of species	Intraspecific variation (N)	Intraspecific variation (percentage)
Mammals	40	1	2.50%
Arthropods	96	21	21.88%
Fish	417	50	11.99%
Amphibians	16	4	25.00%
Reptiles	11	0	0.00%
Mollusks	33	0	0.00%
**Total**	**613**	**76**	**12.40%**

45S rDNA

	Number of species	Intraspecific variation (N)	Intraspecific variation (percentage)
Mammals	156	21	13.46%
Arthropods	424	62	14.62%
Fish	479	63	13.15%
Ampibians	39	11	28.21%
Reptiles	72	1	1.39%
Mollusks	54	1	1.85%
**Total**	**1224**	**159**	**12.99%**
