# Supplementary material for: Evolutionary trends in animal ribosomal DNA loci: introduction to a new online database
Source: Chromosoma. 2017 Nov 30;127(1):141–50. doi: 10.1007/s00412-017-0651-8 (PMC5818627; doi:10.1007/s00412-017-0651-8)
Supplement: Supplementary file 5 — (PDF 699 kb) [file 412_2017_651_MOESM4_ESM.pdf]

Supplementary Table S3. Number of rDNA sites per diploid chromosome sets

Title: Evolutionary trends in animal ribosomal DNA loci: introduction to a new online database

Authors: Jana Sochorová[1*], Sònia Garcia[2*], Francisco Gálvez[3], Radka Symonová[4], Aleš Kovařík[1§]

Address: [1]*Institute of Biophysics, Academy of Sciences of the Czech Republic, Brno CZ–61265, Czech Republic.*

[2] *Institut Botànic de Barcelona (IBB-CSIC-ICUB), Passeig del Migdia s/n, 08038 Barcelona, Catalonia, Spain.*

[3]*Bioscripts - Centro de Investigación y Desarrollo de Recursos Científicos, 41012 Sevilla, Andalusia, Spain.*

[4]*Faculty of Science, University of Hradec Kralove, Hradecka 1285, Hradec Kralove CZ-50003, Czech Republic*

Number of chromosomes per diploid set	5S number	Number of chromosomes per diploid set	45S number
24	6	24	10
24	6	48	2
48	2	48	2
19	2	48	2
52	2	48	2
52	2	19	2
50	2	18	3
249	4	52	2
121	2	52	2
116	2	50	2
146	2	37	2
116	2	249	11
262	4	116	7
23	6	146	7
8	2	116	7
48	4	262	11
26	4	29	2
55	2	38	2
48	2	38	2
44	4	23	4
24	6	23	4
24	6	8	2
50	4	38	2
49	2	6	4
38	8	26	2
24	15	28	2
50	34	28	2
46	2	55	2
23	14	48	2
50	8	34	2
48	2	40	2
54	6	24	3
34	6	24	4
42	6	50	2
38	2	24	2
27	4	24	2
54	2	24	2
54	5	24	2
48	2	38	2
48	5	24	2

46	2	50	2
48	2	30	2
48	2	46	2
48	2	50	7
47	2	11	2
48	2	48	2
32	2	54	2
48	2	34	2
56	2	42	2
56	2	24	2
14	4	24	2
46	6	24	2
42	15	24	2
48	3	38	2
50	4	27	2
50	2	28	2
50	4	34	2
50	2	30	2
50	3	98	2
50	4	46	4
36	5	66	2
48	4	24	2
50	2	54	2
47	4	54	2
50	10	48	8
50	2	48	6
50	10	46	6
50	2	48	22
48	8	48	5
50	4	48	4
50	6	47	2
48	7	48	18
36	4	56	2
50	2	62	2
16	2	32	4
48	2	32	6
56	2	48	2
48	2	46	2
48	2	46	2
38	2	46	2
50	2	46	2
52	2	30	6
48	4	30	7
48	3	31	4
46	4	31	6
24	6	31	6
48	2	56	6
48	2	14	2
8	2	46	2
32	4	18	6

32	4	12	2
24	2	56	6
50	2	176	4
50	2	180	5
50	2	40	2
50	4	42	5
50	2	48	2
50	2	68	2
50	2	50	2
52	2	50	5
26	2	50	2
26	4	50	2
50	2	50	2
42	9	50	5
12	2	36	2
56	8	48	4
24	18	50	2
24	4	47	6
78	2	50	4
18	2	50	2
50	4	50	22
48	2	50	3
48	2	50	7
48	3	50	4
159	3	48	9
100	4	36	4
100	11	50	6
21	2	16	4
50	2	48	2
24	14	56	2
64	6	22	2
48	2	48	2
48	2	48	2
48	18	176	4
48	4	176	4
38	10	30	4
58	2	38	6
48	2	56	2
48	2	32	2
48	2	50	2
48	2	52	2
50	2	48	2
104	54	48	2
56	4	46	2
54	2	16	4
38	2	24	4
46	2	29	2
48	5	30	2
54	4	29	2
54	4	29	2

24	2	16	2
38	2	30	2
20	2	48	2
20	2	60	7
80	2	43	2
80	2	43	2
80	5	43	2
76	2	24	2
80	2	28	2
48	4	60	5
90	2	60	6
46	2	58	7
48	2	60	8
48	2	60	10
78	2	46	6
20	4	32	2
20	4	48	2
48	4	48	2
48	6	32	2
44	2	32	4
48	42	24	2
50	5	50	2
15	3	52	6
42	12	26	2
54	2	26	4
54	2	50	10
54	2	20	8
54	3	22	2
54	2	20	2
100	3	50	2
50	2	42	2
14	2	68	2
20	2	12	2
20	2	42	2
14	4	56	3
20	7	78	6
48	2	36	8
48	2	18	2
48	2	50	2
48	2	48	2
20	2	48	2
18	2	48	2
18	2	159	6
18	2	100	4
18	2	68	2
18	2	22	2
18	2	21	2
18	2	21	2
18	2	57	2
18	2	20	4

18	2	40	2
18	2	80	2
18	2	80	2
18	2	56	4
48	2	50	6
28	2	24	2
48	2	64	15
48	4	48	2
27	10	48	2
38	4	48	2
38	2	48	2
8	2	28	4
8	2	38	9
48	2	18	2
10	3	68	4
22	4	58	2
22	7	23	2
38	2	23	3
48	2	48	2
48	2	48	2
48	2	48	4
48	2	28	6
36	2	50	4
53	12	27	2
46	2	104	12
48	2	56	2
48	2	54	2
48	2	38	2
48	2	46	2
18	4	46	2
20	15	48	6
8	2	48	5
24	11	50	4
46	10	62	8
80	2	54	6
38	2	54	6
48	2	24	2
48	2	38	2
44	4	34	2
4	12	20	6
49	2	20	13
46	2	43	2
50	2	80	6
52	2	80	40
48	2	80	9
16	2	76	2
37	2	48	8
54	28	22	4
54	34	90	3
40	4	46	2

52	4	48	2
54	38	48	2
40	2	78	6
48	4	20	2
48	2	20	2
49	4	20	2
50	4	20	2
46	2	20	2
44	15	20	2
53	4	48	2
56	2	22	10
58	2	32	2
58	10	32	2
58	4	36	4
62	6	36	2
58	2	48	2
44	2	48	2
50	2	37	2
48	2	44	2
24	2	48	2
38	2	50	2
48	4	15	14
27	2	46	2
46	2	42	6
48	2	54	2
50	7	54	2
40	2	54	2
82	4	54	2
62	8	54	2
116	2	28	4
52	4	100	2
52	4	46	5
66	8	50	8
72	9	30	4
42	2	14	7
48	2	20	2
19	2	14	6
48	2	20	5
48	2	14	7
38	4	30	2
48	6	30	2
104	4	30	2
48	2	30	2
44	2	31	6
44	16	30	8
48	6	31	4
50	4	25	8
50	2	28	2
50	6	50	8
50	6	20	7

50	3	32	2
50	6	48	2
25	24	48	2
48	6	48	2
38	2	48	2
18	2	14	2
18	20	14	2
17	23	20	2
18	21	37	2
17	6	18	2
24	2	18	2
24	4	18	4
39	4	18	2
47	4	18	4
50	4	18	2
56	2	18	6
54	2	18	2
58	2	18	2
50	68	18	2
50	74	18	3
50	2	18	2
54	2	18	2
44	14	24	2
18	2	32	2
48	2	48	6
50	8	18	4
36	2	28	2
34	2	48	2
36	2	48	4
48	2	16	3
48	1	59	2
28	2	69	2
48	2	45	4
58	2	55	4
54	3	18	4
54	2	27	10
54	3	38	2
54	2	38	2
54	3	38	2
54	4	8	2
54	4	46	2
54	2	39	2
21	2	37	4
21	2	42	2
50	6	34	2
50	6	38	2
50	7	38	4
52	3	22	2
23	6	80	4
32	2	48	2

48	2	50	2
48	2	10	2
48	2	22	8
48	2	38	2
47	2	38	2
42	5	38	2
38	2	38	2
21	2	27	2
54	14	41	2
44	2	60	4
34	2	36	2
80	2	36	2
42	2	36	2
44	2	48	2
48	2	48	2
50	4	48	3
54	2	36	2
46	2	50	2
48	2	64	8
66	2	20	16
38	2	48	10
50	2	36	2
50	9	53	12
14	4	44	2
14	4	46	2
24	2	38	6
48	2	48	2
48	2	48	2
40	2	48	2
40	2	29	14
26	2	29	9
40	2	29	2
58	2	48	2
54	4	24	2
54	4	20	4
54	2	39	8
56	2	43	2
54	2	43	2
52	2	8	2
26	8	10	4
28	6	14	2
50	2	12	2
38	2	24	12
50	3	24	4
48	2	20	2
54	2	70	8
54	2	22	2
36	2	22	2
48	2	22	2
14	2	46	5

65	2	80	2
80	2	18	2
90	2	38	2
24	2	46	2
24	4	48	4
24	6	48	2
50	4	6	2
50	2	8	2
51	3	6	3
17	18	32	2
18	6	50	2
17	8	4	6
54	2	49	2
42	2	46	2
66	6	50	2
59	4	52	3
68	13	16	2
20	2	36	2
50	2	92	6
50	2	92	6
44	5	48	2
44	6	42	2
44	6	20	5
24	18	42	2
50	4	36	2
56	8	40	2
48	9	42	2
48	2	42	2
48	2	37	2
46	2	54	2
50	3	54	2
50	6	52	2
36	2	40	2
23	16	26	2
47	2	48	2
58	2	48	2
48	2	48	2
48	2	48	6
54	2	48	10
54	3	49	6
54	2	50	6
48	2	36	4
48	2	46	2
48	2	44	8
38	2	37	2
38	2	37	2
32	5	37	2
38	6	37	2
50	16	37	3
23	2	37	4

20	2	37	2
21	2	37	3
22	4	37	2
22	2	30	2
50	16	37	2
50	20	30	2
52	4	53	2
54	4	56	2
46	2	58	2
46	5	50	5
48	3	58	2
58	2	58	2
46	2	62	2
46	2	58	2
56	4	28	2
54	4	50	2
56	6	18	2
56	6	48	2
48	2	24	4
14	5	27	4
36	2	38	4
36	2	46	2
48	2	34	2
44	2	27	2
102	2	46	10
56	2	48	2
54	2	50	13
12	4	40	8
54	3	82	2
54	2	62	3
54	2	116	6
14	2	44	2
24	2	52	3
50	4	52	3
50	4	50	7
50	2	50	12
56	2	38	2
56	2	66	5
44	2	68	8
48	2	75	2
50	6	68	2
48	2	72	2
48	2	73	4
27	6	42	2
26	3	48	2
96	4	19	2
26	2	24	2
26	2	24	2
26	6	48	2
26	2	48	2

42	4	48	2
48	2	36	2
58	3	38	2
56	4	48	2
24	11	104	4
54	18	48	2
50	2	44	4
48	2	32	3
54	2	44	4
64	8	48	2
46	10	50	2
68	9	50	2
55	10	50	2
54	10	50	2
22	10	50	2
38	2	50	2
38	2	25	2
50	4	54	2
50	3	52	2
58	2	62	2
80	2	48	2
80	2	26	3
80	2	26	2
78	7	8	4
78	4	8	9
84	5	8	3
82	7	8	2
84	6	8	4
48	2	8	4
48	2	8	2
48	2	8	2
48	7	8	2
48	2	8	2
54	14	8	2
54	16	8	12
50	4	8	2
52	4	6	6
52	6	8	2
52	8	8	2
60	2	8	6
60	2	8	2
60	2	8	2
24	6	8	2
50	8	8	5
50	5	38	2
51	18	20	4
50	16	16	4
46	3	18	4
50	18	17	6
50	5	18	10

54	4	18	6
54	4	18	4
54	2	17	6
54	4	17	4
54	4	18	6
54	4	18	2
54	4	17	5
42	4	17	4
56	2	24	2
46	2	24	2
48	4	50	2
23	24	36	2
23	22	56	2
46	2	24	6
46	2	54	2
38	2	58	2
38	2	62	22
50	6	18	2
75	6	32	4
17	2	32	4
48	4	32	4
48	2	32	4
56	2	32	4
56	2	32	4
54	2	32	3
17	10	32	4
38	2	32	4
60	2	32	4
23	2	32	4
44	7	32	4
64	4	32	4
38	2	32	6
54	2	32	4
37	2	32	4
23	4	32	2
24	3	32	4
15	4	32	6
30	2	32	4
42	2	32	4
42	2	32	4
46	2	32	10
101	6	32	8
44	6	32	9
40	2	32	6
100	2	32	6
100	2	32	4
100	4	32	4
100	2	32	2
100	2	32	4
100	3	32	4

100	2
48	2
48	2
48	4
56	2
46	2
48	2
46	2
58	2
42	2
52	2
52	2
52	10
52	2
52	2
52	2
52	2
24	2
23	2
36	10
46	2
96	8
40	2
40	2
38	2
38	2
38	2
38	2
34	2
36	2
24	4
48	2
23	2

32	2
32	2
32	10
32	8
32	4
28	2
24	2
50	2
50	2
28	2
54	2
50	6
44	2
54	4
18	2
74	6
164	4
40	2
14	2
20	2
28	2
48	4
37	2
50	2
34	4
30	4
36	2
34	2
36	2
44	3
48	2
48	1
28	2
48	2
58	3
56	2
54	2
54	2
54	2
54	2
54	6
120	2
90	2
52	2
69	4
21	2
21	2
18	2
20	2
20	2

36	2
23	2
168	6
50	2
50	2
50	2
34	2
42	2
52	3
52	2
48	2
23	6
30	2
30	2
34	4
34	4
64	3
12	2
12	2
48	4
48	2
48	5
48	4
47	2
48	2
48	2
48	2
48	2
48	2
20	2
42	2
38	2
10	4
8	2
40	4
46	8
38	4
21	2
54	12
62	2
56	2
36	2
34	2
80	2
42	2
50	2
26	2
44	2
44	2
44	2

54	2
46	2
48	8
50	6
66	2
38	2
32	2
30	2
50	2
50	9
48	4
48	2
34	2
32	2
24	4
14	2
14	2
38	6
48	2
24	2
48	2
48	2
48	2
38	4
7	4
40	28
40	40
40	32
40	26
40	16
40	36
40	38
36	2
40	8
36	6
40	12
36	14
18	8
18	4
40	8
40	20
40	12
40	22
40	12
48	42
26	6
40	10
40	6
40	16
8	2

28	2
58	7
44	8
44	8
54	2
56	2
54	2
52	2
28	6
26	3
28	4
28	8
12	2
50	2
38	2
50	3
48	2
54	2
36	2
58	4
37	2
18	6
34	2
36	3
22	2
38	2
50	12
46	2
62	4
36	2
48	2
70	4
40	2
22	2
36	2
14	4
65	4
80	24
90	4
48	3
24	2
24	2
24	4
50	5
50	2
51	3
25	6
17	17
18	14
17	2

18	2
54	2
42	2
74	2
66	2
59	2
20	7
37	2
37	2
37	2
37	2
37	6
8	2
6	2
8	3
10	2
36	2
36	5
36	2
36	2
36	2
22	3
22	2
22	2
44	2
44	6
44	6
44	6
56	2
60	2
53	2
24	4
50	2
62	10
54	9
30	2
48	4
28	2
26	2
46	2
50	2
50	6
23	4
48	2
47	2
46	2
46	2
38	4
18	8
17	8

52	2
32	10
32	2
32	2
32	2
58	2
54	2
48	2
48	2
54	2
54	2
37	2
37	2
48	2
48	2
48	2
38	4
38	2
66	2
36	2
26	2
48	9
32	4
38	2
50	2
23	2
20	7
26	2
34	2
21	2
32	2
22	2
24	2
26	2
26	4
26	3
26	3
26	2
26	2
56	2
73	2
20	2
20	2
32	2
32	2
32	2
22	3
22	3
14	2
50	12

50	2
52	6
50	2
54	4
30	2
50	2
52	2
46	2
46	4
48	3
58	2
46	2
46	2
56	2
54	2
56	2
56	2
30	2
30	2
30	2
28	4
30	2
30	2
48	2
23	8
28	2
28	2
32	8
32	4
32	4
32	2
32	2
32	2
32	2
32	2
32	4
32	2
32	4
32	2
32	4
32	2
32	4
32	4
32	2
32	2
32	4
32	2
32	4
32	4
32	2

32	2
32	2
32	4
32	4
32	2
32	4
32	2
32	4
32	4
32	2
32	4
32	4
32	4
32	8
46	2
14	8
90	2
46	2
36	2
36	2
8	4
8	4
44	2
102	2
56	2
54	2
13	2
54	3
54	2
54	2
14	2
50	2
37	2
37	2
56	2
56	2
63	8
48	2
48	2
48	2
50	2
38	6
48	42
48	2
27	6
98	6
96	16
26	2
26	2
26	2

26	2
42	6
38	6
58	2
56	2
24	6
24	2
24	2
24	2
50	2
48	6
48	2
34	2
48	2
54	4
64	2
46	2
68	2
55	2
54	8
22	4
36	2
38	2
38	2
68	2
26	2
50	2
50	2
58	2
80	9
80	2
80	2
78	3
84	30
82	2
84	12
80	2
48	4
48	4
30	2
32	2
26	2
34	2
22	2
56	4
24	2
24	2
40	8
48	3
55	2

28	2
48	2
62	2
72	2
54	2
54	2
50	4
52	2
52	7
60	9
60	8
60	12
24	6
24	2
24	4
24	4
50	2
50	2
51	2
50	2
46	3
50	2
50	3
54	54
52	4
42	2
38	4
37	7
26	16
56	2
24	2
24	2
46	2
48	2
66	2
23	4
23	6
46	2
46	2
62	2
62	2
62	3
38	4
38	2
50	6
75	6
17	18
54	2
48	2
48	2

56	2
56	2
54	4
54	2
17	2
18	2
37	2
14	2
14	2
52	2
32	2
56	2
48	2
48	2
30	2
30	2
38	6
46	2
60	3
60	4
60	3
23	2
22	5
44	2
52	7
44	5
48	2
64	4
64	5
48	5
38	2
18	2
54	2
37	4
23	2
24	3
15	4
32	10
30	4
42	4
42	2
28	2
46	2
36	2
24	2
22	4
6	2
6	2
16	2
34	2

16	2
100	6
100	4
100	6
100	4
100	6
100	7
100	10
12	4
50	2
48	2
48	2
48	4
30	2
24	2
24	2
24	2
56	2
24	2
47	2
40	2
48	2
22	2
22	4
22	2
22	2
22	2
22	2
22	2
22	2
22	2
22	2
22	2
22	2
22	2
22	3
22	2
22	2
22	2
25	2
22	2
22	3
22	2
22	2
22	2
12	5
54	2
42	4
42	2
38	2

52	3
52	5
52	4
52	3
52	3
52	3
52	4
52	6
24	3
24	4
24	4
23	4
50	2
50	5
36	2
78	4
78	6
44	4
46	4
22	2
22	3
96	2
38	8
36	8
34	2
22	4
24	4
26	8
30	4
30	2
40	2
40	2
40	2
38	2
38	2
38	2
38	2
74	6
50	2
50	2
50	2
32	2
36	2
24	2
18	2
59	10
59	2
59	9
59	8
59	5

59	2
47	4
57	4
59	4
49	4
57	6
57	6
59	8
59	2
60	4
63	10
23	2
28	4
28	4
56	2
