# Supplementary material for: Evolutionary trends in animal ribosomal DNA loci: introduction to a new online database
Source: Chromosoma. 2017 Nov 30;127(1):141–50. doi: 10.1007/s00412-017-0651-8 (PMC5818627; doi:10.1007/s00412-017-0651-8)
Supplement: Supplementary file 6 — (PDF 461 kb) [file 412_2017_651_MOESM5_ESM.pdf]

Supplementary Table S4. List of karyotypes with known numbers of both 45S and 5S sites (data sets to Supplementary Fig. S1).

Title: Evolutionary trends in animal ribosomal DNA loci: introduction to a new online database
Authors: Jana Sochorová[1*], Sònia Garcia[2*], Francisco Gálvez[3], Radka Symonová[4], Aleš Kovařík[1§]
Address: [1]*Institute of Biophysics, Academy of Sciences of the Czech Republic, Brno CZ–61265, Czech Republic.*
[2] *Institut Botànic de Barcelona (IBB-CSIC-ICUB), Passeig del Migdia s/n, 08038 Barcelona, Catalonia, Spain.*
[3]*Bioscripts - Centro de Investigación y Desarrollo de Recursos Científicos, 41012 Sevilla, Andalusia, Spain.*
[4]*Faculty of Science, University of Hradec Kralove, Hradecka 1285, Hradec Kralove CZ-50003, Czech Republic*

			Percentage
Number of karyotypes assessed		536	
Total number of karyotypes with the same number of 45S and 5S loci		227	42%

			Percentage
Total number of karyotypes with the same number of 45S and 5S loci		227	
Karyotypes with	2 sites	210	92.51%
Karyotypes with	3 sites	1	0.44%
Karyotypes with	4 sites	11	4.85%
Karyotypes with	5 sites	1	0.44%
Karyotypes with	6 sites	4	1.76%

Data set

5S number	45S number
6	10
2	2
2	2
2	2
2	2
2	2
4	11
2	7
2	7
2	7
4	11
6	4
2	2
4	2
2	2
2	2
6	3
6	4
4	2
8	2
15	2
34	2
2	2
8	7
2	2
6	2

6	2
6	2
2	2
4	2
2	2
5	2
2	8
5	6
2	6
2	22
2	5
2	4
2	2
2	18
2	4
2	2
2	6
4	2
6	2
15	5
3	2
4	2
2	5
4	2
2	2
3	2
4	5
5	2
4	4
2	2
4	6
2	4
2	3
4	7
6	4
7	9
4	4
2	6
2	4
2	2
2	2
2	2
2	6
2	2
2	2
4	2
3	2
4	2
6	2
2	2

2	2
4	2
4	4
2	2
2	2
2	6
2	2
4	4
2	2
9	2
2	2
8	3
2	6
2	2
4	2
2	2
2	2
3	2
3	6
11	4
2	2
2	6
14	2
2	2
2	2
18	2
4	2
10	9
2	2
2	2
2	2
2	4
2	4
54	12
4	2
2	2
2	2
2	2
5	5
4	6
4	6
2	2
2	2
2	6
2	13
2	6
5	9
2	2
4	8
2	3

2	2
2	2
2	2
2	6
4	2
4	2
4	2
6	2
2	2
42	2
5	2
3	14
12	6
2	2
2	2
2	2
3	2
2	2
3	2
2	8
2	7
2	2
2	5
4	7
7	7
2	2
2	2
2	2
2	2
2	2
2	2
2	2
2	4
2	2
2	4
2	2
2	6
2	2
2	2
2	2
2	3
2	2
2	2
2	6
2	2
2	2
4	4
10	10
4	2
2	2

2	2
2	2
3	2
2	2
2	2
2	2
2	3
2	2
12	12
2	2
2	2
2	2
2	2
2	2
15	4
2	2
11	12
10	5
2	2
2	2
2	4
2	2
12	6
2	2
2	2
2	2
2	3
2	2
2	2
28	2
34	2
4	2
2	2
4	6
2	10
4	6
4	6
2	2
15	8
4	2
2	2
2	2
10	2
4	2
6	2
2	2
2	2
2	2
2	4
2	4

2	2
2	10
2	2
7	13
2	8
4	2
8	3
2	6
4	3
4	3
8	5
9	2
2	2
2	2
2	2
2	2
2	2
4	2
6	2
4	4
2	2
2	4
16	4
6	2
4	2
2	2
6	2
6	2
3	2
6	2
24	2
6	2
2	2
2	4
20	10
23	4
21	2
6	4
2	2
4	2
2	2
2	2
2	2
68	2
74	2
2	6
2	4
2	2
2	4
8	2

2	2
2	2
2	2
2	2
1	1
2	2
2	2
2	3
3	2
2	2
2	2
3	2
2	6
2	2
2	2
6	2
6	2
7	2
3	3
6	6
2	4
2	2
2	5
2	4
2	2
2	4
2	2
14	12
2	2
2	2
2	2
4	2
2	2
2	2
2	8
2	2
2	2
2	2
9	9
4	2
4	2
2	2
2	2
2	2
2	22
2	12
2	6
2	6
2	7
2	2

2	2
2	2
2	2
8	3
6	4
2	2
2	2
3	3
2	2
2	2
2	2
2	2
2	4
2	4
2	24
2	4
2	2
4	2
6	4
4	5
2	2
3	3
18	17
6	14
8	2
2	2
2	2
6	2
4	2
5	6
6	6
6	6
18	4
4	2
9	4
2	2
3	2
6	6
16	4
2	2
2	2
2	2
2	2
3	2
2	2
2	2
2	2
2	2
2	4
2	2

5	4
6	2
16	2
2	2
2	7
2	2
4	3
2	3
16	12
20	2
4	6
4	4
2	2
5	4
3	3
2	2
2	2
2	2
4	2
4	2
6	2
6	2
2	2
5	8
2	2
2	2
2	2
2	2
2	2
2	2
3	3
2	2
2	2
2	2
2	2
2	2
2	2
2	2
6	2
2	42
2	2
6	6
4	16
2	2
2	2
6	2
2	2
4	6
3	2
4	2

11	6
2	2
2	2
2	4
8	2
10	2
9	2
10	2
10	8
10	4
2	2
2	2
4	2
3	2
2	2
2	9
2	2
2	2
7	3
5	30
7	2
6	12
2	4
2	4
7	3
2	2
14	2
16	2
4	4
4	2
8	7
2	9
2	8
2	12
6	4
8	2
5	2
18	2
16	2
3	3
18	2
5	3
2	54
4	2
2	2
2	2
4	2
24	4
22	6
2	2

2	2
2	4
2	2
6	6
6	6
2	18
4	2
2	2
2	2
2	2
2	2
10	2
2	6
2	4
2	2
7	2
4	4
2	2
2	2
2	4
4	2
3	3
4	4
2	4
2	4
2	2
2	2
2	6
2	4
4	6
2	4
2	6
3	7
2	10
2	2
2	2
4	4
2	2
2	4
2	3
2	5
10	4
2	3
2	3
2	3
2	4
2	3
10	2
2	4
8	2

2	2
2	2
2	2
2	2
2	2
2	2
7	4
2	2
4	2
2	2
