# Supplementary material for: Evolutionary trends in animal ribosomal DNA loci: introduction to a new online database
Source: Chromosoma. 2017 Nov 30;127(1):141–50. doi: 10.1007/s00412-017-0651-8 (PMC5818627; doi:10.1007/s00412-017-0651-8)
Supplement: Supplementary file 7 — (PDF 443 kb) [file 412_2017_651_MOESM6_ESM.pdf]

Supplementary Table S5. Chromosome positions of rDNA loci in different groups of species

Title: Evolutionary trends in animal ribosomal DNA loci: introduction to a new online database

Authors: Jana Sochorová[1*], Sònia Garcia[2*], Francisco Gálvez[3], Radka Symonová [4], Aleš Kovařík[1§]

Address: [1]*Institute of Biophysics, Academy of Sciences of the Czech Republic, Brno CZ–61265, Czech Republic.*

[2] *Institut Botànic de Barcelona (IBB-CSIC-ICUB), Passeig del Migdia s/n, 08038 Barcelona, Catalonia, Spain.*

[3]*Bioscripts - Centro de Investigación y Desarrollo de Recursos Científicos, 41012 Sevilla, Andalusia, Spain.*

[4]*Faculty of Science, University of Hradec Kralove, Hradecka 1285, Hradec Kralove CZ-50003, Czech Republic*

	Number of rDNA loci							
	fish	arthropods	mammals	mollusks	reptiles	birds	amphib.	annelids
5s								
interstitial	145	30	12	18	2	2	6	0
centromeric	177	53	10	5	7	5	9	0
terminal	207	25	22	13	4	2	7	1
45s								
interstitial	104	116	28	9	16		10	2
centromeric	98	159	44	10	4		15	2
terminal	365	197	98	39	25	4	15	6
microchromosome					26	12		

5S			
Position	all species	vertebrates	invertebrates
interstitial	215	167	48
centromeric	266	208	58
terminal	281	242	39
45S			
Position	all species	vertebrates	invertebrates
interstitial	285	158	127
centromeric	332	161	171
terminal	749	507	242
microchromosome	38	38	

45S + 5S	all species
interstitial	500
centromeric	598
terminal	1030
microchromosome	38
