# Supplementary material for: Evolutionary trends in animal ribosomal DNA loci: introduction to a new online database
Source: Chromosoma. 2017 Nov 30;127(1):141–50. doi: 10.1007/s00412-017-0651-8 (PMC5818627; doi:10.1007/s00412-017-0651-8)
Supplement: Supplementary file 8 — (PDF 447 kb) [file 412_2017_651_MOESM7_ESM.pdf]

Supplementary Table S6. Statistical comparison of rDNA positions in chromosomes (Mann Whitney U-test)

Title: Evolutionary trends in animal ribosomal DNA loci: introduction to a new online database
Authors: Jana Sochorová[1*], Sònia Garcia[2*], Francisco Gálvez[3], Radka Symonová[4], Aleš Kovařík[1§]
Address: [1]*Institute of Biophysics, Academy of Sciences of the Czech Republic, Brno CZ–61265, Czech Republic.*
[2] *Institut Botànic de Barcelona (IBB-CSIC-ICUB), Passeig del Migdia s/n, 08038 Barcelona, Catalonia, Spain.*
[3]*Bioscripts - Centro de Investigación y Desarrollo de Recursos Científicos, 41012 Sevilla, Andalusia, Spain.*
[4]*Faculty of Science, University of Hradec Kralove, Hradecka 1285, Hradec Kralove CZ-50003, Czech Republic*

Pairwise comparisons between the groups using the Mann-Whitney U test. The null hypothesis predicted that the proportions of each position are the same. Green, blue and red cells indicated distal, interstitial and proximal positions, respectively. Values with $p<0.05$ were considered as significant (asterisks).

45S (above diagonal) / 5S (below diagonal)

	Fish	Arthropods	Mammals	Mollusks	Orthopotera	Coleoptera
Fish		<0.001*	0.020*	1	<0.001*	0.817
	-	0.018*	0.665	0.745	0.527	0.020*
		<0.001*	0.134	0.78	<0.001*	0.035*
Arthropods	0.003*		0.073	0.013*	<0.001*	<0.001*
	1	-	0.036*	0.163	0.328	<0.001*
	0.002*		<0.001*	<0.001*	<0.001*	<0.001*
Mammals	0.192	0.004*		0.244	<0.001*	0.067*
	1	1	-	1	0.366	0.105
	0.211	0.003*		0.256	<0.001*	0.004
Molluscs	0.018*	<0.001*	0.475		<0.001*	0.962
	0.009*	0.027*	0.063*	-	0.499	0.304
	0.864	0.193	0.308		<0.001*	0.347
Orthoptera	0.011*	0.953	0.006*	<0.001*		<0.001*
	0.398	0.551	0.652	0.157	-	0.011*
	<0.001*	0.347	<0.001*	0.043*		<0.001*
Coleoptera	<0.001*	<0.001*	<0.001*	<0.001*	0.001*	
	0.002*	0.003*	0.009*	<0.001*	0.001*	-
	<0.001*	0.084	<0.001*	0.014*	0.321	

Table frame labels: 45S (top and right), 5S (left and bottom).
