# Supplementary material for: Evolutionary trends in animal ribosomal DNA loci: introduction to a new online database
Source: Chromosoma. 2017 Nov 30;127(1):141–50. doi: 10.1007/s00412-017-0651-8 (PMC5818627; doi:10.1007/s00412-017-0651-8)
Supplement: Supplementary file 9 — (PDF 438 kb) [file 412_2017_651_MOESM8_ESM.pdf]

Supplementary Table S7. Relationship between rDNA distribution and chromosome morphology in insects

Title: Evolutionary trends in animal ribosomal DNA loci: introduction to a new online database
Authors: Jana Sochorová[1*], Sònia Garcia[2*], Francisco Gálvez[3], Radka Symonová[4], Aleš Kovařík[1§]
Address: [1]*Institute of Biophysics, Academy of Sciences of the Czech Republic, Brno CZ–61265, Czech Republic.*
[2] *Institut Botànic de Barcelona (IBB-CSIC-ICUB), Passeig del Migdia s/n, 08038 Barcelona, Catalonia, Spain.*
[3]*Bioscripts - Centro de Investigación y Desarrollo de Recursos Científicos, 41012 Sevilla, Andalusia, Spain.*
[4]*Faculty of Science, University of Hradec Kralove, Hradecka 1285, Hradec Kralove CZ-50003, Czech Republic*

Coleoptera — rDNA position

Typical chromosome morphology in karyotypes	interstitial	pericentromeric	terminal
a/t	0	0	0
m	4	9	51
m+a	0	6	10

Orthoptera — rDNA position

Typical chromosome morphology in karyotypes	interstitial	pericentromeric	terminal
a/t	10	90	14
m	0	0	1
m+a	15	11	10

Coleoptera + *Orthoptera* — rDNA position

Typical chromosome morphology in karyotypes	interstitial	pericentromeric	terminal
a/t	10	90	14
m	4	9	52
m+a	15	17	20

*a - acrocentric, t- telocentric, m- metacentric,
