# Supplementary material for: Evolutionary trends in animal ribosomal DNA loci: introduction to a new online database
Source: Chromosoma. 2017 Nov 30;127(1):141–50. doi: 10.1007/s00412-017-0651-8 (PMC5818627; doi:10.1007/s00412-017-0651-8)
Supplement: Supplementary file 10 — (PDF 594 kb) [file 412_2017_651_MOESM9_ESM.pdf]

Supplementary Table S8. List of centromerically positioned 45S rDNA with corresponding chromosome morphologies

Title: Evolutionary trends in animal ribosomal DNA loci: introduction to a new online database

Authors: Jana Sochorová[1*], Sònia Garcia[2*], Francisco Gálvez[3], Radka Symonová [4], Aleš Kovařík[1§]

Address: [1]*Institute of Biophysics, Academy of Sciences of the Czech Republic, Brno CZ–61265, Czech Republic.*

[2] *Institut Botànic de Barcelona (IBB-CSIC-ICUB), Passeig del Migdia s/n, 08038 Barcelona, Catalonia, Spain.*

[3]*Bioscripts - Centro de Investigación y Desarrollo de Recursos Científicos, 41012 Sevilla, Andalusia, Spain.*

[4]*Faculty of Science, University of Hradec Kralove, Hradecka 1285, Hradec Kralove CZ-50003, Czech Republic*

Summary

	Number (percentage)	Single NOR	Percentage of all karyotypes
Number of karyotypes with centromeric 45S sites (a subset in which chromosome morphology could be distinguished). Multiplicity of rDNA was not considered.	234 (100)		17
Number of karyotypes with centromeric 45S sites in acrocentric chromosomes	182 (78)		14
Number of karyotypes with centromeric 45S sites in metacentric chromosomes	42 (18)	29 (12%) single locus	3 (2)
Number of karyotypes with centromeric 45S sites in both acrocentric and metacentric chromosomes	10 (4)		0.7

Note, metacentric and sumetacentric were counted together, acrocentric and telocentric were counter together

DATA set

Number	Group	Genus	Specific epithet	Chromosome type Acrocentric	Chromosome type Metacentric	Both chr. types	Reference
**1**	Fish	Acaronia	nassa	1	0		162
**2**	Mollusk	Adamussium	colbecki	1	0		258
**3**	Arthropod	Aedes	aegypti	0	1		465
**4**	Fish	Agonostomus	monticola	1	0		168

**5**	Arthropod	Amblytropidia	sp.	1	0		261
**6**	Mammal	Antrozous	pallidus	1	0		395
**7**	Fish	Argyrosomus	regius	1	0		158
**8**	Fish	Artedidraco	skottsbergi	1	0		358
**9**	Flatworm	Aspidogaster	limacoides	1	0		325
**10**	Fish	Astatotilapia	latifasciata	1	0		174, 239
**11**	Fish	Baryancistrus	xanthellus	0	1		424
**12**	Fish	Bathygobius	sp.	1	0		425
**13**	Arthropod	Brasilacris	gigas	1	0		236
**14**	Mammal	Carollia	castanea	1	0		24
**15**	Mammal	Carollia	perspicillata	1	0		24
**16**	Flatworm	Caryophyllaeides	fennica	0	1		182
**17**	Arthropod	Cauratettix	borelli	1	0		452
**18**	Mollusk	Clausinella	fasciata	0	1		364
**19**	Fish	Cobitis	sp.	1	0		298
**20**	Fish	Cobitis	taenia	1	0		44
**21**	Fish	Conger	conger	1	0		4
**22**	Fish	Coris	julis	1	1	1	110
**23**	Fish	Corydoras	britskii	1	0		189
**24**	Fish	Cryodraco	atkinsoni	0	1		219
**25**	Arthropod	Cycloptiloides	americanus	1	0		337
**26**	Arthropod	Descampsacris	serrulatum	1	0		84
**27**	Mammal	Desmodus	rotundus	0	1		192
**28**	Fish	Dicentrarchus	labrax	1	0		86
**29**	Fish	Dicentrarchus	punctatus	1	0		86
**30**	Arthropod	Dichotomius	mundus	0	1		193
**31**	Fish	Diplodus	sargus	1	0		194
**32**	Fish	Dissostichus	eleginoides	1	0		195
**33**	Fish	Dissostichus	mawsoni	1	0		195
**34**	Arthropod	Dociostaurus	jagoi	1	0		206
**35**	Arthropod	Dociostaurus	maroccanus	1	0		88, 206
**36**	Fish	Economidichthys	pygmaeus	1	0		80
**37**	Fish	Eleginops	maclovinus	1	0		196

**38**	Mollusk	Ensis	arcuatus	1	0		198
**39**	Arthropod	Enyaliopsis	ephippiatus	1	0		431
**40**	Fish	Epinephelus	awoara	1	0		114
**41**	Mammal	Eptesicus	fuscus	1	0		395
**42**	Arthropod	Eugasteroides	loricatus	1	0		431
**43**	Arthropod	Eumastusia	koebelei	1	0		447
**44**	Arthropod	Eumigus	monticola	1	0		453
**45**	Arthropod	Eurytoma	robusta	0	1		331
**46**	Arthropod	Eurytoma	serratulae	0	1		331
**47**	Flatworm	Fasciola	hepatica	1	0		380
**48**	Fish	Gymnocypris	chui	1	0		457
**49**	Fish	Gymnocypris	scleracanthus	1	0		457
**50**	Annelid	Haemopis	sanguisuga	0	1		210
**51**	Fish	Haplochromis	obliquidens	1	1	1	7
**52**	Arthropod	Heteracris	adspersa	1	0		88
**53**	Fish	Hoplerythrinus	unitaeniatus	1	1	1	459
**54**	Fish	Hoplias	malabaricus	0	1		51
**55**	Amphibian	Hypsiboas	semilineatus	0	1		217
**56**	Mollusk	Chamelea	gallina	0	1		364
**57**	Fish	Channa	punctatus	0	1		8
**58**	Mammal	Choeroniscus	godmani	1	0		395
**59**	Mammal	Choeronycteris	mexicana	1	0		395
**60**	Arthropod	Chorthippus	bigguttulus	1	1	1	206
**61**	Arthropod	Chromacris	nuptialis	1	0		206, 236
**62**	Arthropod	Chromacris	speciosa	1	0		206, 236
**63**	Arthropod	Isophya	altaica	1	0		311
**64**	Arthropod	Isophya	amplipennis	1	0		311
**65**	Arthropod	Isophya	andreevae	1	0		311
**66**	Arthropod	Isophya	armena	1	0		311
**67**	Arthropod	Isophya	autumnalis	1	0		311
**68**	Arthropod	Isophya	brunneri	1	0		311
**69**	Arthropod	Isophya	burechi	1	0		311
**70**	Arthropod	Isophya	camptoxypha	1	0		311

**71**	Arthropod	Isophya	gulae	1	0		311
**72**	Arthropod	Isophya	hospodar	1	0		311
**73**	Arthropod	Isophya	kraussii	1	0		311
**74**	Arthropod	Isophya	longicaudata	1	0		311
**75**	Arthropod	Isophya	longicaudata	1	0		311
**76**	Arthropod	Isophya	major	1	0		311
**77**	Arthropod	Isophya	miksici	1	0		311
**78**	Arthropod	Isophya	modestior	1	0		311
**79**	Arthropod	Isophya	nervosa	1	0		311
**80**	Arthropod	Isophya	obtusa	1	0		311
**81**	Arthropod	Isophya	pavelii	1	0		311
**82**	Arthropod	Isophya	pienensis	1	0		311
**83**	Arthropod	Isophya	plevnensis	1	0		311
**84**	Arthropod	Isophya	rectipennis	1	0		311
**85**	Arthropod	Isophya	rhodopensis	1	0		311
**86**	Arthropod	Isophya	rhodopensis	1	0		311
**87**	Arthropod	Isophya	rhodopensis	1	0		311
**88**	Arthropod	Isophya	rizeensis	1	0		311
**89**	Arthropod	Isophya	schneideri	1	0		311
**90**	Arthropod	Isophya	speciosa	1	0		311
**91**	Arthropod	Isophya	stenocauda	1	0		311
**92**	Arthropod	Isophya	stenocauda	1	0		311
**93**	Arthropod	Isophya	strubei	1	0		311
**94**	Arthropod	Isophya	sureyai	1	0		311
**95**	Arthropod	Isophya	taurica	1	0		311
**96**	Arthropod	Isophya	thracica	1	0		311
**97**	Arthropod	Isophya	tosevski	1	0		311
**98**	Arthropod	Isophya	yaraligozi	1	0		311
**99**	Arthropod	Isophya	zernovi	1	0		311
**100**	Amphibian	Itapotihyla	langsdorffii	1	0		366
**101**	Fish	Labeo	rohita	1	0		289
**102**	Lamprey	Lampetra	zanandreai	0	1		392
**103**	Mammal	Laonastes	aenigmamus	1	1	1	469

**104**	Mammal	Lasiurus	cinereus	1	0	395
**105**	Fish	Lepidonotothen	nudifrons	1	0	357
**106**	Fish	Lepidonotothen	squamifrons	1	0	357
**107**	Arthropod	Leptopilina	boulardi	1	0	492
**108**	Lamprey	Lethenteron	camtschaticum	0	1	468
**109**	Fish	Leuciscus	cephalus	1	0	130
**110**	Fish	Leuciscus	idus	1	0	130
**111**	Fish	Leuciscus	leuciscus	1	0	130
**112**	Mammal	Lonchophylla	mordax	1	0	395
**113**	Reptile	Masticophis	flagellum	1	0	234
**114**	Mammal	Microtus	agrestis	1	0	24
**115**	Mammal	Monophyllus	redmani	1	0	395
**116**	Fish	Mugil	incilis	1	0	31
**117**	Mammal	Mus	cervicolor	1	0	375
**118**	Mammal	Mus	cooki	1	0	375
**119**	Mammal	Mus	cypriacus	1	0	375
**120**	Mammal	Mus	famulus	1	0	375
**121**	Mammal	Mus	haussa	1	0	375
**122**	Mammal	Mus	hortulanus	1	0	393
**123**	Mammal	Mus	indutus	1	0	375
**124**	Mammal	Mus	matthey	1	0	375
**125**	Mammal	Mus	minutoides	1	0	375
**126**	Mammal	Mus	musculoides	1	0	375
**127**	Mammal	Mus	musculus	1	0	393
**128**	Mammal	Mus	musculus	1	0	375.376
**129**	Mammal	Mus	musculus	1	0	375.376
**130**	Mammal	Mus	musculus	1	0	393, 394
**131**	Mammal	Mus	musculus	1	0	15, 55, 394
**132**	Mammal	Mus	musculus	1	0	375.376
**133**	Mammal	Mus	pahari	1	0	375
**134**	Mammal	Mus	platythrix	1	0	55
**135**	Mammal	Myotis	keyasi	1	0	395
**136**	Arthropod	Nocaracris	cyanipes	1	0	454

**137**	Fish	Notothenia	coriiceps	1	0		266
**138**	Fish	Ocyurus	chrysurus	1	0		68
**139**	Fish	Ompok	bimaculatus	1	0		29
**140**	Fish	Ompok	pabda	1	0		29
**141**	Fish	Oncorhynchus	masou	1	1	1	45, 269
**142**	Annelid	Ophryotrocha	diadema	1	0		227
**143**	Amphibian	Oreobates	barituensis	0	1		489
**144**	Amphibian	Oreobates	berdemenos	0	1		489
**145**	Amphibian	Oreobates	discoidalis	0	1		489
**146**	Arthropod	Paranocaracris	rubripes	1	0		454
**147**	Arthropod	Parapyrrhicia	acutilobata	1	0		491
**148**	Arthropod	Parapyrrhicia	diamantina	1	0		491
**149**	Fish	Pargus	auriga	1	0		194
**150**	Fish	Pargus	pargus	1	0		194
**151**	Amphibian	Phasmahyla	spectabilis	0	1		164
**152**	Amphibian	Phyllomedusa	bahiana	1	0		164
**153**	Amphibian	Phyllomedusa	bahiana	1	0		493
**154**	Amphibian	Phyllomedusa	distincta	1	0		493
**155**	Amphibian	Phyllomedusa	nordestina	1	0		164
**156**	Amphibian	Phyllomedusa	tarsius	1	0		493
**157**	Amphibian	Phyllomedusa	vaillantii	1	0		493
**158**	Arthropod	Podisma	pedestris	1	0		402
**159**	Reptile	Podocnemis	expansa	0	1		496
**160**	Reptile	Podocnemis	unifilis	0	1		496, 514
**161**	Arthropod	Poecilimon	affinis	1	0		311
**162**	Arthropod	Poecilimon	ampliatus	1	0		311
**163**	Arthropod	Poecilimon	anatolicus	1	0		311
**164**	Arthropod	Poecilimon	armeniacus	1	0		311
**165**	Arthropod	Poecilimon	ataturki	1	0		311
**166**	Arthropod	Poecilimon	bischoffi	1	0		311
**167**	Arthropod	Poecilimon	bosphoricus	1	0		311
**168**	Arthropod	Poecilimon	brunneri	1	0		311
**169**	Arthropod	Poecilimon	celebi	1	0		311

**170**	Arthropod	Poecilimon	cervus	1	0		311
**171**	Arthropod	Poecilimon	ebneri	1	0		311
**172**	Arthropod	Poecilimon	ersisi	1	0		311
**173**	Arthropod	Poecilimon	glandifer	1	0		311
**174**	Arthropod	Poecilimon	heinrichi	1	0		311
**175**	Arthropod	Poecilimon	chopardi	1	0		311
**176**	Arthropod	Poecilimon	jablanicensis	1	0		311
**177**	Arthropod	Poecilimon	karakushi	1	0		311
**178**	Arthropod	Poecilimon	klisuriensis	1	0		311
**179**	Arthropod	Poecilimon	ledereri	1	0		311
**180**	Arthropod	Poecilimon	macedonicus	1	0		311
**181**	Arthropod	Poecilimon	maritinae	1	0		311
**182**	Arthropod	Poecilimon	marmaraensis	1	0		311
**183**	Arthropod	Poecilimon	miramae	1	0		311
**184**	Arthropod	Poecilimon	orbelicus	1	0		311
**185**	Arthropod	Poecilimon	ornatus	1	0		311
**186**	Arthropod	Poecilimon	pechevi	1	0		311
**187**	Arthropod	Poecilimon	pliginskii	1	0		311
**188**	Arthropod	Poecilimon	roseoviridis	1	0		311
**189**	Arthropod	Poecilimon	serratus	1	0		311
**190**	Arthropod	Poecilimon	schmidtii	1	0		311
**191**	Arthropod	Poecilimon	similis	1	0		311
**192**	Arthropod	Poecilimon	toros	1	0		311
**193**	Arthropod	Poecilimon	turcicus	1	0		311
**194**	Arthropod	Poecilimon	ukrainicus	1	0		311
**195**	Arthropod	Poecilimon	zonatus	1	0		311
**196**	Arthropod	Poecilimon	zwicki	1	0		311
**197**	Fish	Prochilodus	nigricans	0	1		63
**198**	Arthropod	Pycnogaster	cucullata	1	1	1	21
**199**	Cartilaginous fis	Raja	asterias	1	0		270
**200**	Cartilaginous fis	Raja	montagui	1	1	1	66
**201**	Mammal	Rattus	rattus	1	0		375.377
**202**	Arthropod	Rhammatocerus	brasiliensis	1	0		30, 206, 261

**203**	Arthropod	Rhammatocerus	brunneri	1	0		261
**204**	Arthropod	Rhammatocerus	palustris	1	0		261
**205**	Arthropod	Rhammatocerus	pictus	1	0		261
**206**	Fish	Rhomboplites	aurorubens	1	0		68
**207**	Fish	Rineloricaria	capitonia	0	1		498
**208**	Fish	Rineloricaria	latirostris	0	1		498
**209**	Mammal	Rousettus	aegyptiacus	0	1		395
**210**	Fish	Salmo	salar	0	1		45, 302, 303
**211**	Amphibian	Scinax	auratus	0	1		333
**212**	Amphibian	Scinax	eurydice	0	1		333
**213**	Fish	Semaprochilodus	insignis	0	1		63
**214**	Fish	Semaprochilodus	taeniurus	0	1		63
**215**	Arthropod	Shirakiacris	shirakii	1	0		202
**216**	Fish	Synbranchus	marmoratus	1	1	1	312, 323
**217**	Fish	Tanakia	limbata	1	0		262
**218**	Arthropod	Tapinoma	nigerrimum	0	1		276
**219**	Mammal	Taterillus	sp.	0	1		277
**220**	Mammal	Taterillus	tranieri	0	1		277
**221**	Fish	Tetraodon	fluviatilis	1	0		140
**222**	Arthropod	Thisoicetrinus	pterostichus	1	0		202
**223**	Fish	Tor	putitora	1	1	1	87
**224**	Fish	Trachydoras	paraguayensis	0	1		507
**225**	Fish	Trichomycterus	brasiliensis	0	1		346
**226**	Amphibian	Triturus	vulgaris	0	1		27
**227**	Fish	Umbra	pygmaea	0	1		280
**228**	Mammal	Uroderma	bilobatum	0	1		395
**229**	Mammal	Uroderma	magnirostrum	1	0		395
**230**	Reptile	Varanus	komodoensis	0	1		466
**231**	Arthropod	Warramaba	picta	0	1		28
**232**	Arthropod	Warramaba	virgo	0	1		28
**233**	Arthropod	Xestotrachelus	robustus	1	0		236, 282
**234**	Arthropod	Xyleus	discoideus	1	0		30
